# Orchestrating the gut microbiota–mitochondrial–immune axis in gynecological diseases: mechanisms and dual-targeting therapeutic strategies

**DOI:** 10.3389/frph.2026.1845581

**Published:** 2026-07-01

**Authors:** Haixia Tang, Yiting Zhang, Yanting Wang, Ze Zhou, Rong Sun, Rong Chen, Lijuan Yang, Mengqiu Shao, Jiabao Liao

**Affiliations:** 1 The First Clinical Medical College, Yunnan University of Chinese Medicine, Kunming, Yunnan, China; 2 The Second Clinical Medical College, Yunnan University of Chinese Medicine, Kunming, Yunnan, China; 3 Medical Affairs Department, Jiaxing Hospital of Traditional Chinese Medicine, Jiaxing, Zhejiang, China

**Keywords:** endometriosis, gynecological malignancies, microbiota–mitochondria axis, mitophagy/PGC-1α, NLRP3/cGAS–STING, polycystic ovary syndrome, premature ovarian insufficiency, reproductive endocrinology

## Abstract

The “gut microbiota–mitochondria axis” has become the core hub connecting the metabolism, immunity, and endocrine regulation of gynecological diseases. In this review, the hierarchical regulation mechanism of this axis is systematically combed: at the upstream level, intestinal short-chain fatty acids (SCFAs), bile acids (BAs), tryptophan derivatives, and other metabolites can activate AMPK/PGC-1α, FXR/TGR5, and AhR-mediated energy sensing and receptor signaling pathways; On the functional level, bacterial lipopolysaccharide-TLR4 signal and cGAS–STING/NLRP3 inflammasome pathway activated by cytoplasmic mitochondrial DNA (mtDNA) can amplify innate immune response; At the effect level, mitochondrial reactive oxygen species (ROS), mitochondrial dynamics, and PINK1/Parkin-mediated mitophagy are the common key nodes to regulate mitochondrial quality and inflammatory response. Combined with the two-way relationship between the estrobolome and steroid production, the above processes together form a self-reinforcing closed loop of “metabolic input-immune amplification-oxidative stress/autophagy-endocrine regulation”. Based on this theoretical framework, this paper analyzes the disease-specific correlations among polycystic ovary syndrome, endometriosis, premature ovarian insufficiency, and gynecological malignancies, and puts forward a dual-targeted treatment idea with research value. The intervention plan with microbiota as the core aims to adjust the metabolite spectrum and endotoxin level; Mitochondria-centered interventions focus on restoring cell energy metabolism and apoptosis sensitivity. In addition, this review constructs a hierarchical research framework of “microbiota-metabolomics-mitochondria” to clarify the targeted phenotypes in the pathway, and provide guidance for subsequent clinical trial design and long-term monitoring. With the deep integration of multi-omics technology and targeted interventions, the gut microbiota–mitochondria axis is expected to become an important breakthrough in precision medical treatment of gynecological diseases and build a brand-new bridge between basic mechanism research and clinical transformation.

## 1 Introduction

Gynecological diseases, such as polycystic ovary syndrome (PCOS), endometriosis, premature ovarian insufficiency (POI), and gynecological malignancies, seriously damage women's fertility and quality of life, and are closely related to endocrine disorders, abnormal energy metabolism, and chronic inflammation (1, 2). In recent years, research on microbiology and mitochondrial biology has been continuously promoted. Many studies have supported that the interaction between gut microbiota and mitochondria is a common pathological mechanism in the occurrence and development of various gynecological diseases (3, 4). Gut microbiota has been described as the “second genome” of the human body, which can produce short-chain fatty acids (SCFAs), bile acids (BAs), indole derivatives, trimethylamine N-oxide (TMAO) and other metabolites (5, 6). It is noteworthy that the gut microbiota also constitute an “estrobolome”, that is, a collection of bacterial genes that regulate the level of estrogens in the whole body through the deconjugation and recycling of estrogens (7, 8). These metabolites can modulate the energy metabolism and redox homeostasis of mitochondria, thereby regulating the processes of cell apoptosis, immune homeostasis, and hormone synthesis (3, 9). At the same time, mitochondria are the core of cellular energy generation and stress signal transduction, and abnormal mitochondrial function will change the redox and inflammatory environment in cells, which in turn affects the composition and stability of gut microbiota (10, 11). This two-way regulatory relationship constitutes the “gut microbiota–mitochondria axis”, and its role in metabolic and nervous system diseases has been increasingly recognized (12, 13), and research in the field of gynecological diseases has gradually attracted attention.

Existing research suggests that there is gut microbiota dysbiosis in PCOS patients, which reduces the production of SCFAs, aggravates endotoxin leakage, and further exacerbates mitochondrial oxidative stress and insulin resistance (14, 15). In patients with endometriosis, the inflammatory reaction induced by lipopolysaccharide and mitochondrial DNA (mtDNA) released from damaged mitochondria can jointly activate the NLRP3 inflammasome and form a chronic inflammatory microenvironment (16, 17). Microbial metabolite-induced mitochondrial apoptosis and metabolic reprogramming in patients with POI and gynecological malignancies lead to a decline in ovarian reserve function and tumor drug resistance, respectively (18–20). The above results propose that the interaction between microbiota and mitochondria is not a product of local lesions but a key phenotypic change in the progression of gynecological diseases. However, most current studies focus on gut microbiota dysbiosis or mitochondrial damage alone, and rarely integrate their related mechanisms, which not only limits the understanding of the pathogenesis of gynecological diseases but also hinders the development of targeted treatment programs.

**Figure 1:**
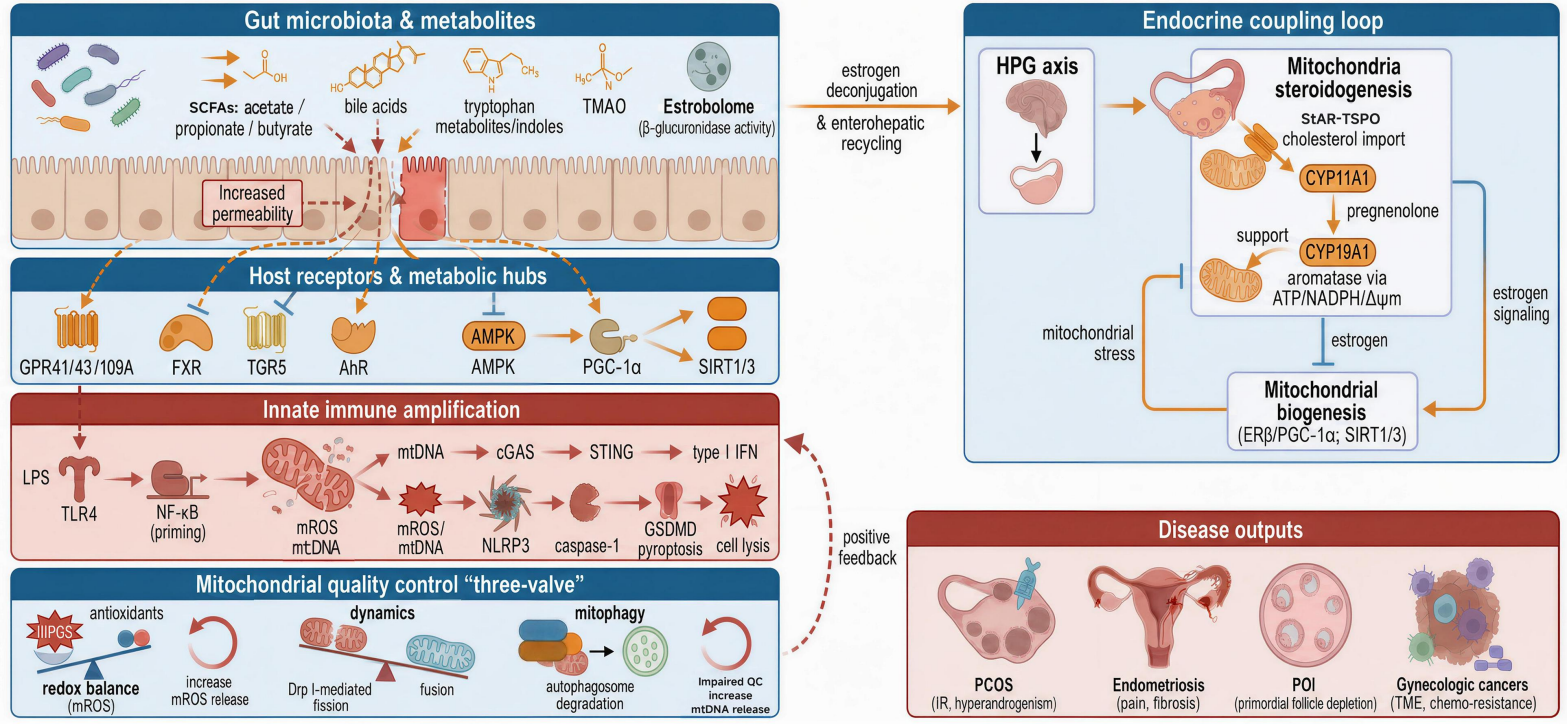
Gut microbiome–mitochondria–immune–endocrine axis in gynecologic diseases. Gut dysbiosis reshapes microbial metabolites (SCFAs, bile acids, indoles, and TMAO) and may increase intestinal permeability, thereby elevating systemic exposure to LPS. These signals interact with host receptors and metabolic hubs (GPR41/43/109A, FXR, TGR5, and AhR) and converge on AMPK–PGC-1α and sirtuin pathways to regulate mitochondrial biogenesis, metabolic adaptation, and redox balance. LPS–TLR4–NF-κB signaling provides inflammatory priming, whereas mitochondrial stress-associated signals (mROS and mtDNA release) may further amplify innate immune activation through NLRP3 inflammasome signaling (caspase-1–mediated GSDMD pyroptosis) and cGAS–STING–type I IFN pathways. Mitochondrial quality control mechanisms, including antioxidant defense systems, mitochondrial dynamics, and PINK1/Parkin-mediated mitophagy, may function as regulatory “brakes”, while impairment of these processes may reinforce a positive feedback loop between inflammation and mitochondrial damage. An endocrine coupling loop links estrobolome-dependent estrogen recycling with the HPG axis and mitochondrial steroidogenesis, thereby contributing to disease-specific phenotypes in PCOS, endometriosis, POI, and gynecologic malignancies. Solid arrows indicate mechanisms supported by relatively consistent human and experimental evidence, whereas dashed arrows represent pathways that are predominantly supported by preclinical, indirect, or emerging evidence. Inhibitory T-shaped lines indicate regulatory or suppressive effects. SCFAs, short-chain fatty acids; TMAO, trimethylamine N-oxide; LPS, lipopolysaccharide; mROS, mitochondrial reactive oxygen species; mtDNA, mitochondrial DNA; AMPK, AMP-activated protein kinase; PGC-1*α*, peroxisome proliferator-activated receptor-gamma coactivator-1 alpha; HPG, hypothalamic–pituitary–gonadal; PCOS, polycystic ovary syndrome; POI, premature ovarian insufficiency; TME, tumor microenvironment.

From the perspective of research methods, this paper is a narrative review, aiming to build an integrated theoretical framework. By Integrating evidence from population-based observational cohorts, animal models, and *in vitro* mechanistic studies, we summarize the basic biological laws of the interaction between gut microbiota and mitochondria. As illustrated in Figure 1, we further analyze the specific processes of this regulator*y* axis across PCOS, endometriosis, POI, and gynecological malignancies. Critically, we also examine the current status of human clinical interventional evidence to date, identifying the limitations and translational challenges in this field. The purpose of this paper is to put forward a verifiable hypothesis for translational research and to provide new ideas for the study of the gut microbiota–mitochondria axis.

## 2 Biological basis of gut microbiota–mitochondria interplay

The interaction between gut microbiota and mitochondria is not unidirectional conduction, but rather forms a two-way coupling system through metabolites, receptor signals, and immune regulation networks (21, 22). On the one hand, intestinal SCFAs, secondary BAs, indole compounds and other metabolites can regulate mitochondrial energy metabolism, oxidative stress and apoptosis (23, 24); On the other hand, excessive production of ROS, membrane potential depolarization and mtDNA cytoplasmic leakage caused by abnormal mitochondrial function will destroy epithelial hypoxia and change the mucosal immune state, and then reshape intestinal microbial composition (9, 11). This mutually regulated “gut microbiota–mitochondria axis” is a core pathological hub of metabolic disorder and chronic inflammation in gynecological diseases (1, 25). This section outlines the biological basis of gut microbiota–mitochondria interactions from three perspectives: microbial metabolites in host systemic signaling, microbiota-driven immune activation and amplification within the tissue microenvironment, and mitochondrial function and homeostasis.

### 2.1 Microbial metabolites in host systemic signaling

Intestinal microbial metabolites can regulate energy metabolism pathways, receptor signaling, oxidative stress reactions, and autophagy processes, promote mitochondrial biogenesis, maintain mitochondrial functional homeostasis, and achieve effective communication between exogenous nutritional signals and endocrine regulation (26, 27). SCFAs are the main energy source for colonic epithelial cells; they can participate in the tricarboxylic acid cycle, activate intracellular energy-sensing pathways, improve energy utilization efficiency and insulin sensitivity, and promote mitochondrial biogenesis and fatty acid oxidation (28). At the same time, SCFAs can act on G protein-coupled receptors such as GPR41, GPR43, and GPR109A, and regulate intestinal hormone secretion and systemic metabolic rhythm (29).

Similarly, the secondary BAs produced by gut microbiota can act as ligands for FXR and TGR5 receptors, regulating mitochondrial membrane potential, ROS generation, and systemic energy homeostasis (30). In addition to metabolic regulation, these receptor-mediated signals also affect estrogen synthesis and ovarian function. Gut-derived microbial enzymes, such as β-glucuronidase, regulate the ratio of active to inactive estrogens in the circulation through the estrobolome (8, 31), and then affect the mitochondrial respiratory capacity and antioxidant defense function of the reproductive system, suggesting the relationship between intestinal microbial activity and mitochondrial energy metabolism (18). Tryptophan metabolites, especially indole derivatives, can regulate the activity and antioxidant capacity of respiratory chain complexes, and the kynurenine pathway can also act as a metabolic regulator to modulate mitophagy and biogenesis (24, 32). To summarize, existing studies have supported that microbial metabolites can indirectly transmit exogenous nutritional signals to the endocrine network, while maintaining the stability of mitochondrial quality and function.

### 2.2 Microbiota-driven immune activation and amplification

In addition to metabolic regulation, an immune amplification loop can explain how gut microbiota and mitochondria jointly regulate chronic inflammation in the tissue microenvironment. After endotoxin translocation caused by intestinal barrier damage, pathogen-associated molecular patterns (PAMPs) such as lipopolysaccharide (LPS) will activate innate immune receptors (33), providing a key initiating signal for the activation of the NLRP3 inflammasome (34, 35).

At the same time, ROS and mtDNA released from damaged mitochondria, as endogenous damage-associated molecular patterns (DAMPs), cannot be effectively eliminated; instead, they promote the assembly of the NLRP3 inflammasome and the release of pro-inflammatory factors (36, 37), further amplifying the initial inflammatory response induced by microbiota (38).

Exogenous PAMPs and endogenous DAMPs work together to form a continuous excessive inflammatory cycle (39). In endometriosis and other diseases, this kind of synergistic immune cascade reaction is considered an important contributing factor that triggers a vicious circle of pain, local inflammation, and fibrosis (17, 40, 41).

### 2.3 Mitochondrial regulation and homeostasis

Mitochondrial quality control is key to avoiding excessive inflammation and metabolic disorder (42). Stress-induced electron leakage and inflammatory activation of the electron transport chain will first increase the production of ROS, and beneficial metabolites such as SCFAs can alleviate this problem by strengthening the antioxidant system (43). However, excessive accumulation of ROS beyond the scavenging capacity will accelerate lipid peroxidation and eventually lead to the collapse of the mitochondrial membrane potential (44).

Mitochondrial dynamics also plays an important role. Under inflammatory and oxidative conditions, excessive mitochondrial fission leads to structural fragmentation, decreased ATP production, and impaired steroid production, which results in abnormal follicular development (45); Conversely, enhanced mitochondrial fusion and cristae remodeling help maintain functional stability (46).

Finally, mitophagy can specifically remove damaged mitochondria, prevent mtDNA and ROS from being released into the cytoplasm, and block the vicious circle caused by NLRP3 inflammasome activation (47). Damage to this clearance pathway will not only aggravate the inflammatory reaction but also lead to defects in tissue repair and drug resistance (48).

To sum up, the interaction between microbiota and mitochondria is reflected in three interrelated levels: systemic metabolic mediation, microenvironmental immune amplification, and mitochondrial homeostasis maintenance, which together constitute a “metabolism-immunity-endocrine” regulatory network. The intracellular signaling pathways and molecular targets that play regulatory roles will be elaborated in detail later.

## 3 Intracellular signaling and molecular integration of the gut microbiota–mitochondria axis

### 3.1 Translating metabolic signals: the SCFAs/AMPK/PGC-1α axis

At the cellular level, intestinal metabolites mainly regulate mitochondrial function through the AMPK/PGC-1α signaling hub. After SCFAs (especially butyrate) enter cells, the intracellular AMP/ATP ratio is altered, and then AMPK is phosphorylated. *In vitro* and *in vivo* studies have supported that activated AMPK can directly phosphorylate PGC-1α, and at the same time activate SIRT1, which deacetylates PGC-1α (49). This synergistic post-translational modification promotes mitochondrial biogenesis, strengthens fatty acid β-oxidation, and regulates cellular energy metabolism (50). Therefore, the SCFAs/AMPK/PGC-1α signaling cascade is a core molecular bridge that alleviates lipotoxicity and an important defense mechanism against metabolic disorders (51).

In the same way, secondary BAs transform microbial signals into metabolic adaptation reactions through signaling pathways mediated by specific receptors. BAs bind to the membrane G protein-coupled receptor TGR5, which increases intracellular cAMP levels and further promotes AMPK activation (52). At the same time, BAs activate the nuclear receptor FXR, regulating lipid metabolism and maintaining the transcription of related target genes (53, 54). Experimental models show that abnormal regulation of the BA-receptor axis leads to mitochondrial energy metabolism disorder, which is also a common molecular phenotype in PCOS and intrahepatic cholestasis of pregnancy (55, 56).

Tryptophan metabolism is another important intracellular regulatory pathway. Microbial indole derivatives can up-regulate the activity of the respiratory chain complex (57), and the kynurenine pathway is a double-edged sword for metabolic regulation. Mechanistic studies show that physiologically relevant concentrations of kynurenine metabolites can maintain mitochondrial number via the AMPK/SIRT1 axis (58, 59), but excessive accumulation of kynurenine disrupts the redox balance and inhibits mitophagy (60). Cohort studies of PCOS and endometriosis patients found that increased kynurenine concentration was positively correlated with disease severity, and that it exacerbates mitochondrial damage through a pathological positive feedback loop (61).

To summarize, various metabolic signals converge on the AMPK/PGC-1*α* and SIRT1 regulatory networks, transforming intestinal microbial changes into long-term transcriptional programs and regulating mitochondrial number and cellular homeostasis.

### 3.2 Innate immune crosstalk: TLR4 and mtDNA-driven cGAS–STING/NLRP3 activation

The synergistic effect of intestinal microbial signals and mitochondrial stress is mainly realized through the convergence of innate immune cascade pathways, and the core follows a “double-hit” activation mode. The first step is exogenous signal transduction. After intestinal barrier damage, intestinal LPS enters the circulation (62). As a typical PAMP, it binds to TLR4 on the surface of macrophages, triggering NF-κB nuclear translocation, providing a key initiating signal, and greatly up-regulating the transcription of NLRP3 inflammasome components and pro-inflammatory cytokine precursors (63, 64).

**Figure 2:**
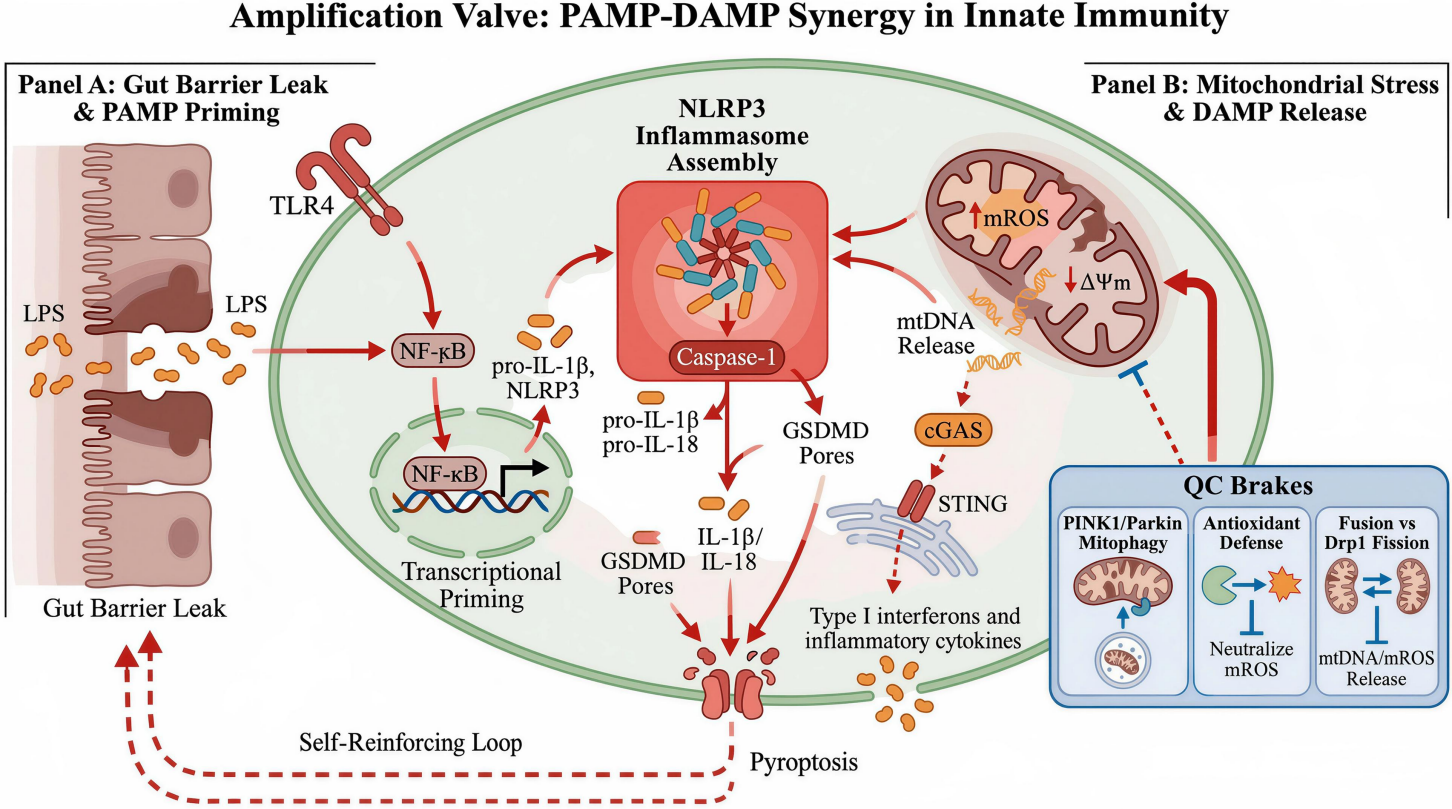
Amplification valve: PAMP–DAMP synergy drives innate immune escalation relevant to gynecologic inflammation. Panel A illustrates gut barrier leak–associated PAMP priming. Increased intestinal permeability may elevate systemic LPS exposure, thereby activating TLR4–NF-κB signaling and promoting transcriptional priming of inflammasome-associated components, including NLRP3 and pro-IL-1β/pro-IL-18. Panel B depicts mitochondrial stress–associated DAMP release. Increased mROS and reduced mitochondrial membrane potential (↓ΔΨm) may promote cytosolic mtDNA release, which can contribute to NLRP3 inflammasome assembly and, in parallel, engage cGAS–STING signaling to induce type I interferons and inflammatory cytokines. Inflammasome activation promotes caspase-1–dependent IL-1β/IL-18 maturation and GSDMD cleavage, leading to pore formation and pyroptosis. Pyroptosis and inflammatory amplification may further aggravate barrier dysfunction and mitochondrial injury, thereby promoting a self-reinforcing inflammatory loop relevant to chronic pelvic inflammation and tissue remodeling. Mitochondrial quality-control (“QC”) mechanisms, including PINK1/Parkin-mediated mitophagy, antioxidant defenses, and balanced fusion–fission dynamics, may function as regulatory “brakes” that limit mtDNA/mROS release and dampen this amplification process. Solid arrows denote well-established mechanisms, whereas dashed arrows indicate emerging, indirect, or predominantly preclinical pathways. T-shaped lines represent inhibition. PAMP, pathogen-associated molecular pattern; DAMP, damage-associated molecular pattern; LPS, lipopolysaccharide; mROS, mitochondrial reactive oxygen species; ΔΨm, mitochondrial membrane potential; mtDNA, mitochondrial DNA; cGAS, cyclic GMP-AMP synthase; STING, stimulator of interferon genes; GSDMD, gasdermin D; QC, quality control.

Subsequently, mitochondrial dysfunction releases endogenous DAMPs, providing key activation signals. Under oxidative stress, the integrity of the mitochondrial membrane is damaged, leading to the leakage of mtDNA and ROS into the cytoplasm (65). *In vitro* experiments with macrophages have shown that free mtDNA in the cytoplasm can be directly recognized by cGAS, which activates the cGAS–STING pathway, induces phosphorylation of IRF3 and NF-κB, and promotes the expression of type I interferons and broad-spectrum inflammatory mediators (66). At the same time, oxidized mtDNA together with mitochondrial ROS can bind to and activate the NLRP3 inflammasome protein, triggering its oligomerization to form an active inflammasome, activating caspase-1, cleaving mature IL-1β and IL-18, and finally causing pyroptosis (47, 67), driving the inflammatory amplification cascade illustrated in Figure 2.

The molecular interaction between the TLR4-NF-κB and mtDNA-cGAS/NLRP3 inflammasome axes forms an irreversible positive feedback cycle of excessive inflammation. The continuous activation of the NLRP3 inflammasome and the sustained release of IL-1β will not only aggravate local tissue damage but also disrupt the adaptive immune response, promote macrophage polarization towards the pro-inflammatory M1 type, overactivate Th17 cells, and inhibit the function of regulatory T cells (68–70). This complex intracellular signaling network is the core molecular basis for the formation of a chronic inflammatory microenvironment and the disruption of immune tolerance in reproductive system diseases (71).

### 3.3 Molecular execution of mitophagy: the PINK1/parkin pathway

Intestinal metabolites and innate immune signals can regulate oxidative stress, and the molecular execution mechanism of mitophagy is the ultimate key to mitochondrial quality control. ROS is not only a downstream injury marker but also an important regulatory factor in the gut microbiota–mitochondria axis regulatory system. Physiological concentrations of ROS can act as signaling molecules to initiate antioxidant defense, but continuous gut microbiota dysbiosis leads to LPS translocation, which causes the oxidative load to far exceed the cellular clearance capacity, leading to severe lipid peroxidation and collapse of the mitochondrial membrane potential (ΔΨm) (72, 73).

*In vitro* mechanistic studies have supported that loss of mitochondrial membrane potential is a critical trigger for the activation of the PINK1/Parkin signaling pathway (74). After mitochondrial depolarization, PINK1 can no longer be degraded by the proteasome but stably accumulates on the outer mitochondrial membrane (75). PINK1 accumulation phosphorylates ubiquitin molecules and recruits the E3 ubiquitin ligase Parkin to damaged mitochondria (76). Activated Parkin ubiquitinates outer mitochondrial membrane proteins to form recognizable ubiquitin chain signals, and then autophagy adaptor proteins bind to the ubiquitinated mitochondrial proteins and connect them with the autophagy protein LC3, thus achieving selective encapsulation of damaged mitochondria and lysosomal degradation (77, 78).

It is worth noting that the integrity of the PINK1/Parkin-mediated clearance pathway determines the cellular immunophenotype. Experimental models show that beneficial microbial metabolites such as SCFAs and hydrogen sulfide (H_2_S) can indirectly support mitophagy and maintain mitochondrial function (79, 80); On the other hand, chronic dysbiosis-induced inflammation will inhibit or damage the PINK1/Parkin pathway, resulting in massive accumulation of damaged mitochondria and release of large amounts of mtDNA and ROS, which may further promote cGAS–STING and NLRP3 inflammasome activation, as illustrated in Figure 2 (81).

Therefore, the PINK1/Parkin axis serves as a key molecular checkpoint that may influence whether transient microbiota-induced stress is resolved or progresses into persistent and potentially irreversible inflammatory lesions (82).

## 4 The role of gut microbiota–mitochondria interaction in gynecological diseases

The interaction between gut microbiota and mitochondria shows specific clinical phenotypes in different gynecological diseases. Table 1 (17, 37, 41, 83–96) summarizes representative alterations in microbiota composition, metabolites, and mitochondrial function across major gynecological diseases, including polycystic ovary syndrome(PCOS), endometriosis, premature ovarian insufficiency (POI), and gynecological malignancies.

**Table 1:** Integrated alterations in gut microbiota, metabolites, and mitochondrial function across gynecological diseases and their immunological consequences.

Disease	Alpha diversity	Taxonomic shifts	Key metabolic alterations	Mitochondrial phenotypes	Immune/pathological outcomes	Key references
PCOS	↓ in most cohorts [H] (Fecal; heterogeneous across BMI subgroups)	↓ *Bifidobacterium*, *Faecalibacterium* prausnitzii [H/A] *Prevotella*: inconsistent across cohorts (↑ and ↓ reported; BMI- and geography-dependent) [H] ↑ *Bacteroides*, *Escherichia*/*Shigella* inconsistent F/B ratio [H]	↓ SCFAs → ↓ insulin sensitivity [H/A] ↑ LPS → systemic endotoxaemia [A] ↓ Secondary bile acids (GDCA, TUDCA) [A]	↑ mROS; ↓ ATP [A/I] Impaired AMPK/PGC-1*α* → ↓ mitochondrial biogenesis [A] ↑ Lipid peroxidation (MDA); ↓ ΔΨmin granulosa cells [A/I]	LPS–TLR4–NF-κB → insulin resistance [H/A] NLRP3 inflammasome activation → ↑ IL-1β, IL-18 [A] Inflammatory hyperandrogenism; chronic low-grade inflammation [H]	(84–87)
Endometriosis	Variable/↓ (fecal) [H] (Fecal and peritoneal microbiomes are compositionally distinct; direct diversity comparison is not valid)	↑ Proteobacteria (*Escherichia coli*, *Klebsiella* spp.) [H] ↓ *Lactobacillus* dominance [H] ↑ *Shigella*/*Escherichia* ratio (severe Endometriosis) [H] MR-confirmed causal genera: *Holdemania*, *Desulfovibrio*, *Haemophilus* [H]	↑ Systemic/peritoneal LPS [H/A] ↓ SCFAs → impaired mucosal immune tolerance [A] ↑ β-glucuronidase → ↑ free oestrogens (estrobolome dysregulation) [H/A]	mROS accumulation; mtDNA cytoplasmic release [A/I] Impaired PINK1/Parkin-mediated mitophagy [A/I] ↓ *ΔΨ*min ectopic stromal cells [I] (*Mainly experimental; limited human in situ evidence*)	NLRP3 inflammasome and cGAS–STING activation (mainly experimental) [A/I] Chronic peritoneal inflammation [H] Fibrosis-associated microenvironment; infertility [H]	(17, 37, 41, 83)
POI	↓ (limited cohorts) [H] (↑ β-diversity is the more consistent finding)	↓ SCFA-producing bacteria (*Faecalibacterium* spp.; heterogeneous across cohorts) [H] ↑ Diet-associated taxa (*Dorea*, *Sutterella*) [H] MR-confirmed causal genera (e.g., *Ruminococcus* spp.) [H] Gut virome dysbiosis co-occurs and modulates bacteriome [H/A]	↑ TMAO (mainly animal evidence; human data limited) [A] ↓ SCFAs [H/A] ↑ β-glucuronidase → estrobolome dysregulation [H/A]	↑ ROS → granulosa cell apoptosis [A/I] ↓ *ΔΨ*min oocytes; impaired mitophagy [A/I] Possible cGAS–STING activation [I] (*Mainly experimental; human oocyte data limited*)	Inflammaging-like phenotype (↑ IL-6, TNF-α) [H/A] Th17/Treg imbalance → autoimmune oophoritis [H/A] Granulosa cell dysfunction → impaired steroidogenesis [A/I] Accelerated follicular depletion [A]	(88–92)
Eynecological malignancies	Inconsistent [H] (Intratumoral and gut microbiomes are distinct compartments; α-diversity cross-comparison is methodologically invalid)	↑ *Fusobacterium nucleatum* (ovarian, endometrial, cervical cancers) [H] Altered Firmicutes/Bacteroidetes balance [H] *F. nucleatum* → TLR4/NF-κB → chemoresistance [A/I] Intratumoral taxa (*Methylobacter*, *Klebsiella*, *Micromonospora*) linked to cervical cancer metastasis [H]	Altered bile acids → impaired FXR/TGR5-mediated tumour suppression [A/I] ↑ Oestrogenic metabolites (↑ β-glucuronidase) [H] ↓ SCFAs → loss of HDAC inhibition → epigenetic pro-tumourigenic dysregulation [I]	Warburg-like metabolic reprogramming (↑ aerobic glycolysis) [H/A/I] ↑ ROS tolerance; impaired apoptosis sensitivity [A/I] ↑ Drp1 dysregulation → tumour proliferation and reduced cisplatin sensitivity [A/I]	CD8+ T-cell dysfunction; M2 macrophage polarisation [H/A] Chemotherapy resistance linked to mitochondrial remodelling [A/I] Microbiota-driven IL-10/TGF-β → immunosuppressive TME [A/I]	(93–96)

[H], human observational/clinical studies; [A], animal studies; [I], *in vitro* mechanistic experiments. Alpha diversity refers to metrics (Shannon index, Chao1) derived from fecal samples in human observational studies. Peritoneal fluid and intratumoral microbiomes are compositionally distinct from fecal microbiota and cannot be directly compared for diversity metrics. Taxonomic shifts represent relative abundance changes and may vary across cohorts due to heterogeneity in study design, population characteristics (BMI, geography, diet), and analytical pipelines. *Prevotella* abundance in PCOS shows inconsistent directionality across cohorts and is not reported as a uniform increase. Mitochondrial phenotypes involving cGAS–STING activation and PINK1/Parkin-mediated mitophagy are primarily supported by experimental models; direct human tissue evidence is limited. TMAO elevation in POI is based mainly on animal model data; human metabolomics evidence remains limited. MR, Mendelian randomization. F/B ratio, Firmicutes/Bacteroidetes ratio.

SCFAs, short-chain fatty acids; BAs, bile acids; LPS, lipopolysaccharide; mROS, mitochondrial reactive oxygen species; ΔΨm, mitochondrial membrane potential; TMAO, trimethylamine N-oxide; mtDNA, mitochondrial DNA; GDCA, glycodeoxycholic acid; TUDCA, tauroursodeoxycholic acid; HDAC, histone deacetylase; FXR, farnesoid X receptor; TGR5, Takeda G-protein-coupled receptor 5; Drp1, dynamin-related protein 1; TME, tumor microenvironment; MDA, malondialdehyde; NLRP3, NLR family pyrin domain-containing protein 3 inflammasome; cGAS–STING, cyclic GMP-AMP synthase–stimulator of interferon genes pathway; PGC-1α, peroxisome proliferator-activated receptor-*γ* coactivator 1α; PINK1/Parkin, PTEN-induced kinase 1/E3 ubiquitin ligase Parkin; MR, Mendelian randomization.

However, when interpreting the above research results, we need to face the inherent heterogeneity of current microbiota research. There are often differences in microbiota signals among different population groups. Such differences are not statistical errors, but an important part of research evidence. Dietary pattern, body mass index, insulin resistance, region, medication history, and other confounding factors will significantly affect the host microbiota (97); Methodological differences such as sequencing processes, analysis methods, and sample type selection will also change the results of taxonomic classification (98). In addition, the mechanisms of cGAS–STING and PINK1/Parkin-mediated mitophagy are mostly based on experimental research, which should be interpreted accordingly. Therefore, the relationships summarized herein should be understood as context-dependent rather than universally applicable.

To ensure the rigor of the research method and avoid exaggerating the clinical readiness, the following sections analyze the research evidence for each disease from three levels: clinical evidence and human observations, animal model mechanism support, and *in vitro* cell studies, and distinguish verified mechanisms and speculative hypotheses from those clinically documented, so as to provide a clear basis for subsequent translational research.

### 4.1 Polycystic ovary syndrome (PCOS)

**Figure 3:**
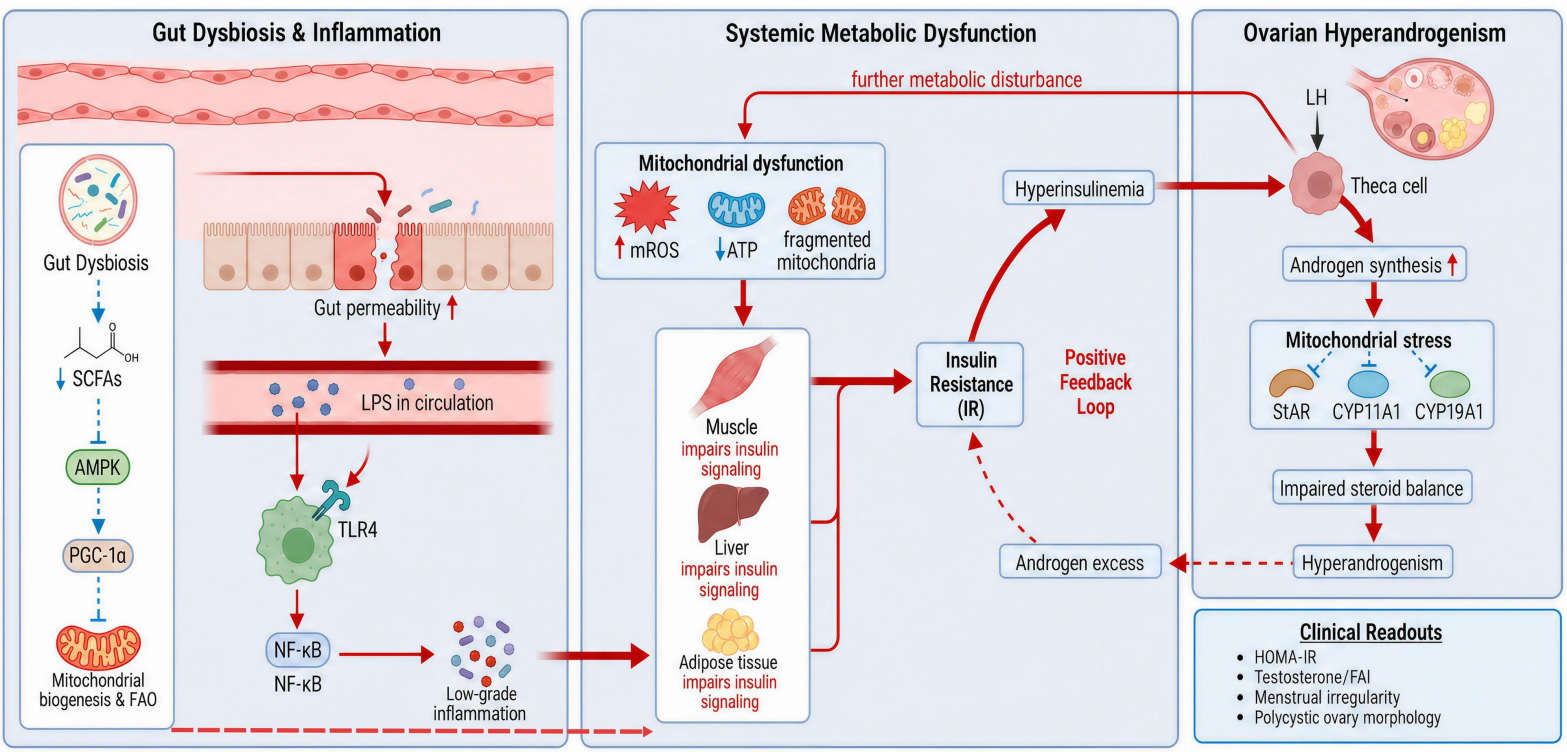
Gut dysbiosis–mitochondria–insulin resistance axis in PCOS-associated hyperandrogenism. Gut dysbiosis reduces SCFA production, potentially weakening AMPK–PGC-1*α* signaling and impairing mitochondrial biogenesis and fatty-acid oxidation (FAO). Increased gut permeability may elevate circulating LPS levels, thereby activating TLR4–NF-κB signaling and contributing to chronic low-grade inflammation. These metabolic and inflammatory inputs converge on mitochondrial dysfunction (↑mROS, ↓ATP, and altered mitochondrial dynamics) and impaired insulin signaling in muscle, liver, and adipose tissue, promoting systemic insulin resistance and hyperinsulinemia. Hyperinsulinemia may synergize with luteinizing hormone (LH) to enhance androgen synthesis in ovarian theca cells, while mitochondrial stress may impair steroidogenic regulation involving StAR, CYP11A1, and CYP19A1, thereby disrupting steroid balance. Excess androgen production may further aggravate metabolic dysfunction, forming a positive feedback loop relevant to PCOS progression. Clinical manifestations include increased HOMA-IR, elevated testosterone/free androgen index (FAI), menstrual irregularity, and polycystic ovarian morphology. Solid arrows indicate relatively well-supported mechanisms, whereas dashed arrows represent indirect or emerging pathways. PCOS, polycystic ovary syndrome; SCFAs, short-chain fatty acids; LPS, lipopolysaccharide; TLR4, Toll-like receptor 4; NF-κB, nuclear factor kappa B; mROS, mitochondrial reactive oxygen species; IR, insulin resistance; LH, luteinizing hormone; StAR, steroidogenic acute regulatory protein; HOMA-IR, homeostatic model assessment for insulin resistance; FAI, free androgen index; FAO, fatty-acid oxidation.

PCOS is a complex endocrine disease, the core features of which are hyperandrogenism, ovulatory dysfunction, and serious metabolic problems such as insulin resistance (99). Figure 3 shows the multi-organ regulatory network related to gut microbiota dysbiosis, systemic metabolic disorder, and ovarian hyperandrogenism.

#### 4.1.1 Clinical evidence and human observations in PCOS

Sequencing data from population cohorts showed that the gut microbiota composition of PCOS patients differed from that of healthy controls, mainly manifested by decreased microbial alpha diversity, reduced SCFA-producing taxa, decreased abundance of beneficial bacteria such as *Bifidobacterium* and *Faecalibacterium prausnitzii*, and altered *Prevotella* abundance across cohorts, which were associated with clinical indicators such as insulin sensitivity, blood lipid levels, and serum androgen concentration (100, 101). Metabolomics analysis also found that PCOS patients had disordered metabolism of circulating secondary BAs and insufficient levels of SCFAs (102).

Regarding clinical interventions, emerging evidence from recent randomized controlled trials (RCTs) and systematic reviews suggests that probiotic and synbiotic supplementation can modulate gut microbial composition and improve insulin resistance and hyperandrogenism in PCOS patients (103, 104). A 2024 RCT further reported a significant reduction in total testosterone following synbiotic intervention combined with lifestyle modification (105). Recent clinical trials are also exploring dietary strategies, including Mediterranean diet interventions, for improving metabolic and endocrine outcomes in PCOS patients (106), while a head-to-head RCT by Borzan et al. indicated that probiotic supplementation significantly improved metabolic and endocrine parameters compared with metformin and placebo (107). However, despite these promising findings, direct clinical evidence linking gut-targeted interventions to restoration of mitochondrial bioenergetics remains lacking. Mechanistic pathways identified in animal models, such as the *Escherichia coli* Nissle 1917–IL-22–mitochondria axis, still await clinical validation (108). Therefore, larger standardized clinical trials are needed to determine whether the proposed gut–microbiota–mitochondrial axis can be functionally modulated in humans (104, 109).

#### 4.1.2 Evidence from animal models of PCOS

Animal models of PCOS further supported the above clinical phenomena at the mechanistic level and suggest that changes in the microbiota may contribute to systemic endocrine disorders. SCFA-producing taxa in the model decreased, which was associated with suppression of the AMPK/PGC-1*α* signaling axis and reduced mitochondrial biogenesis and fatty acid oxidation (110). At the same time, intestinal permeability increased, which promoted LPS to enter the circulation, thereby contributing to endotoxemia, activated the TLR4/NF-κB pathway, and formed a chronic low-grade inflammatory state (111, 112). Inflammation may further aggravate systemic insulin resistance and lead to hyperinsulinemia. Hyperinsulinemia cooperated with luteinizing hormone (LH) to overactivate ovarian theca cells, leading to excessive androgen synthesis, forming a vicious cycle of “insulin resistance-hyperinsulinemia-hyperandrogenism” (112, 113).

#### 4.1.3 Evidence from *in vitro* studies in PCOS

*In vitro* experiments at the cellular level was associated with mitochondria are involved in local ovarian dysfunction. After metabolic or inflammatory stress treatment, granulosa cells and theca cells exhibit obvious mitochondrial energy metabolism disorders, characterized by decreased ATP production, damaged membrane potential, and a large amount of mitochondrial ROS accumulation (102, 114). Excessive mitochondrial ROS directly inhibits cholesterol transport into mitochondria mediated by the STAR protein, reduces the activities of key steroidogenic enzymes such as CYP11A1 and CYP19A1, disrupts the processes of steroid production and follicular maturation, and aggravates the androgen phenotype of PCOS patients (115).

### 4.2 Endometriosis

**Figure 4:**
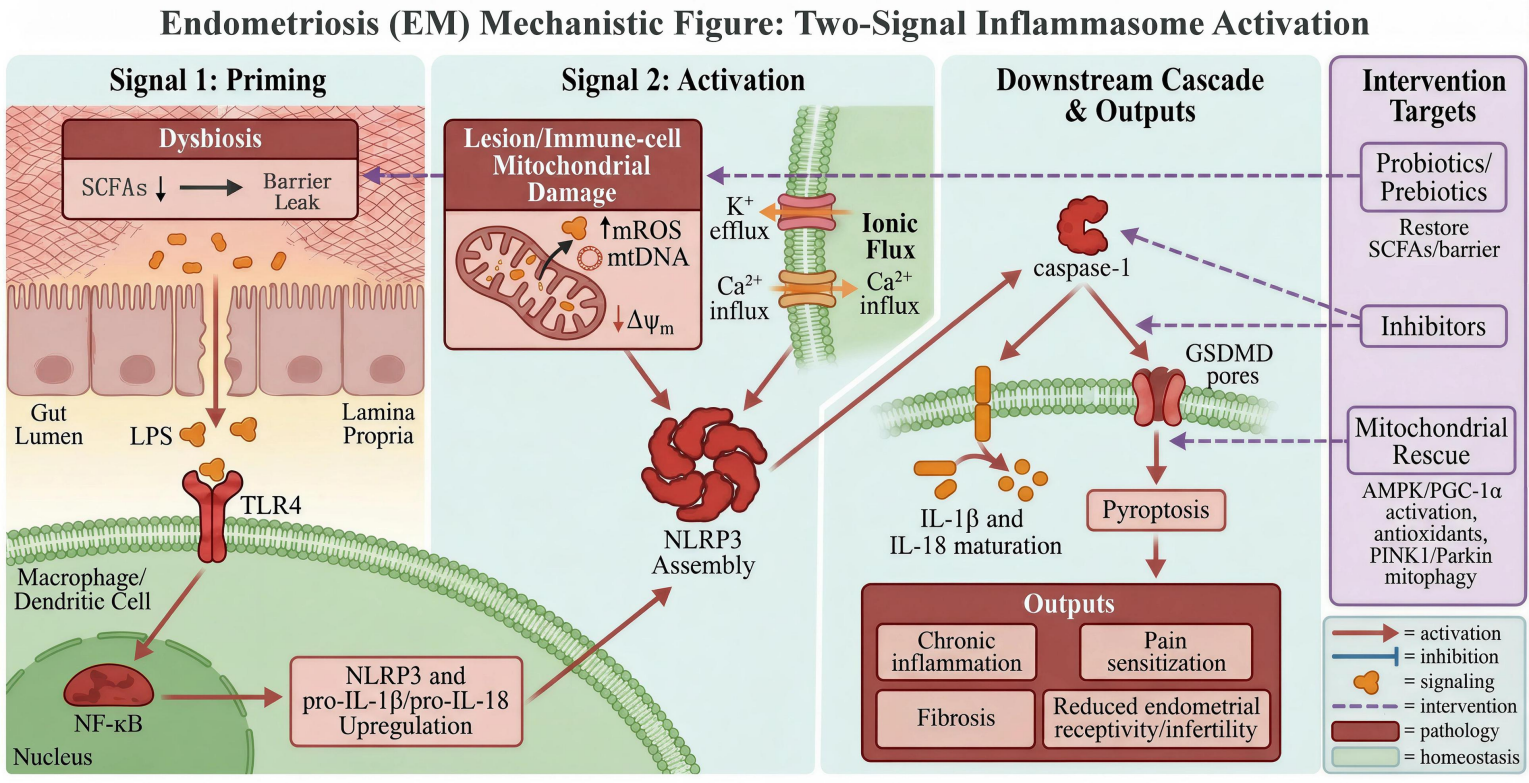
Two-signal inflammasome activation model in endometriosis. Signal 1 (priming): gut dysbiosis with reduced SCFAs and barrier leak increases LPS translocation, activating TLR4–NF-*κ*B signaling in macrophages and dendritic cells and promoting the upregulation of NLRP3 and pro-IL-1β/pro-IL-18. Signal 2 (activation): lesion- and immune-cell–associated mitochondrial damage (↑mROS, ↓ΔΨm, and mtDNA release), together with ionic flux (K^+^ efflux/Ca^2+^ influx), promotes NLRP3 assembly and caspase-1 activation, resulting in IL-1β/IL-18 maturation, GSDMD pore formation, and pyroptosis. These inflammatory events may contribute to chronic inflammation, fibrosis, pain sensitization, and impaired endometrial receptivity associated with infertility. Therapeutic leverage points include microbiota restoration (probiotics/prebiotics), inhibition of the TLR4/NLRP3–caspase-1–GSDMD axis, and mitochondrial rescue strategies involving AMPK–PGC-1α activation and PINK1/Parkin-mediated mitophagy. Solid arrows indicate activation and signaling pathways, whereas dashed arrows represent therapeutic interventions. SCFAs, short-chain fatty acids; LPS, lipopolysaccharide; TLR4, Toll-like receptor 4; NF-κB, nuclear factor kappa B; IL, interleukin; mROS, mitochondrial reactive oxygen species; ΔΨm, mitochondrial membrane potential; mtDNA, mitochondrial DNA; GSDMD, gasdermin D.

Endometriosis is a chronic inflammatory disease. The core pathology is the ectopic growth of endometrial-like tissue, which is often accompanied by dysmenorrhea, chronic pelvic pain, and infertility (116). Existing studies show that the pathogenesis of endometriosis is closely related to gut microbiota dysbiosis, mitochondrial dysfunction, and amplification of the innate immune response in the peritoneal microenvironment, as illustrated in Figure 4 (16).

#### 4.2.1 Clinical evidence and human observations in endometriosis

Clinical cohort studies found that the gut microbiota composition of patients changed significantly, mainly manifested by the proliferation of Proteobacteria (e.g., *Escherichia coli*) and a decrease in dominant protective bacteria such as *Lactobacillus* (117, 118). Recent studies further suggested that estrobolome alterations may contribute to estrogen metabolism imbalance in endometriosis, thereby influencing inflammatory activity and disease progression (119). Microbiota dysbiosis was associated with reduced levels of beneficial SCFAs in the systemic circulation and peritoneal cavity, as well as impaired intestinal barrier integrity and a possible “leaky gut” phenotype. These alterations may contribute to elevated endotoxin (LPS) levels in the circulation and peritoneal fluid, which have been associated with the severity of pelvic inflammation and disease stage (120, 121).

Regarding clinical intervention, evidence remains limited but is gradually emerging. Adjunctive probiotic or synbiotic therapy has been associated with reduced inflammatory markers and improved postoperative pain in endometriosis patients (122). A triple-blind RCT further showed that astaxanthin supplementation enhanced antioxidant capacity while reducing pro-inflammatory cytokines (123). Dietary interventions, including a low-FODMAP regimen, also demonstrated symptomatic improvement in randomized trials (124). However, current clinical evidence remains insufficient to determine whether gut-targeted interventions can directly modulate the peritoneal inflammatory microenvironment or alter disease progression in endometriosis. Recently published trials, including the ProMetrioS study, together with ongoing anti-inflammatory dietary intervention studies, may provide further translational insights into microbiota-based therapeutic strategies (125, 126).

#### 4.2.2 Evidence from animal models of endometriosis

Animal models of endometriosis provide an *in vivo* mechanistic basis for clinical observation. The endometriosis model supports that intestinal endotoxemia is a persistent cause of inflammation in the peritoneal microenvironment, and that LPS continuously activates the TLR4/NF-κB pathway in peritoneal immune cells (127, 128). Experimental studies further suggested that mitochondrial stress-associated mtDNA release may contribute to cGAS–STING activation and amplification of peritoneal inflammation (37). Intervention on the gut-peritoneal axis by depleting the microbiota or blocking TLR4 significantly reduced the volume of ectopic lesions and decreased inflammatory cell infiltration, proving that the synergistic effect of intestinal PAMPs and local inflammation contributes to the colonization and angiogenesis of ectopic endometrial lesions (129).

#### 4.2.3 Evidence from *in vitro* studies in endometriosis

*In vitro* experiments at the cellular level using ectopic endometrial stromal cells and peritoneal macrophages clarified that mitochondrial stress and the NLRP3 inflammasome are the final effector factors of disease regulation (130). The microbial initiation signal aggravates mitochondrial dysfunction, leading to the accumulation of endogenous DAMPs such as decreased membrane potential, excessive production of mitochondrial ROS, and cytoplasmic release of mtDNA. Mechanistic experiments suggested that these endogenous danger signals may promote NLRP3 inflammasome activation, accompanied by caspase-1 activation, Gasdermin D (GSDMD) cleavage, pyroptosis, and increased release of IL-1β and IL-18, thereby contributing to a fibrotic and inflammatory local microenvironment (131). This microbiota-associated mitochondrial inflammatory signaling cascade inhibits ectopic cell apoptosis, aggravates local fibrosis, and increases pain sensitivity, which eventually leads to a decrease in endometrial receptivity associated with infertility in endometriosis patients (132, 133).

### 4.3 Premature ovarian insufficiency (POI)

POI refers to premature depletion of the ovarian follicular pool before the age of 40, leading to gonadotropic hypogonadism and infertility. More and more studies have suppoeted that the gut microbiota–mitochondria axis is key to regulating ovarian lifespan, and that intestinal metabolic disorders amplify systemic inflammation and destroy ovarian mitochondrial quality control (88, 134).

#### 4.3.1 Clinical evidence and human observations in POI

Clinical data show that compared to the healthy population, the intestinal microbiota of POI patients has decreased alpha diversity and altered abundance of specific taxa (131). The increase in circulating microbial metabolites such as TMAO is positively correlated with the decline in ovarian reserve function (88). However, as these observations are largely correlational, it remains to be determined whether gut dysbiosis is a primary driver of POI or a secondary consequence of the altered hormonal environment.

Clinical interventional evidence for POI remains particularly scarce. To date, no completed RCT has directly evaluated microbiota-targeted interventions for restoring ovarian reserve in POI patients; the closest human-level evidence is a Mendelian randomization study identifying genetically predicted causal links between specific gut taxa and POI risk, though the authors explicitly noted that clinical trials are still required to confirm these associations (89). Prospective interventional studies are urgently needed to determine whether gut-targeted approaches could offer a supportive strategy to preserve residual ovarian function in this population (91, 134).

#### 4.3.2 Evidence from animal models of POI

Animal models of POI have provided preliminary mechanistic insights into the interaction between the gut microbiota and ovarian function. These studies showed that microbiota depletion induced by broad-spectrum antibiotics, together with exposure to POI-inducing toxins, was associated with follicular atresia and diminished ovarian reserve (135, 136). At the mechanistic level, microbial metabolites and LPS translocation lead to systemic inflammation, thereby disrupting the follicular microenvironment. Experimental evidence further suggested that mitochondrial stress-associated signaling, including possible mtDNA-mediated cGAS–STING activation, may contribute to inflammatory amplification in the ovarian microenvironment (92). In addition, animal studies suggested that raising circulating SCFA levels can protect follicular development, indicating that microbial metabolic homeostasis can delay premature ovarian failure (137, 138).

#### 4.3.3 Evidence from *in vitro* studies in POI

At the cellular level, the POI disease phenotype may partly stem from the imbalance in the coupling relationship between the estrobolome and steroid production. The steroid production process of granulosa cells is extremely energy-consuming and highly dependent on mitochondrial ATP for energy supply (139). Experiments have supported that microbial metabolites such as TMAO and LPS can induce mitochondrial depolarization, excessive ROS production, and impaired mitophagy (140, 141). These stress factors inhibit cholesterol transport mediated by the StAR protein, down-regulate the expression of CYP11A1 and CYP19A1, and hinder the synthesis of progesterone and estrogen (142). Abnormal mitochondrial-steroid production may form a pathological cycle: PINK1/Parkin-mediated mitophagy is impaired, leading to cytoplasmic release of mtDNA, activation of cGAS–STING inflammatory signaling, and further aggravation of granulosa cell aging and apoptosis (143, 144). Continued activation of cGAS–STING promotes local formation of “inflammaging” in the ovary (92). This mitochondria-related chronic innate immune response induces granulosa cells to secrete aging-related phenotypes and accelerates follicular atresia and primordial follicle pool depletion (145, 146). The gut microbiota–mitochondria axis triggers a sterile inflammatory cascade through mtDNA release, which represents a potential contributing mechanism in ovarian aging in POI patients. To summarize, the joint action of microbiota-induced inflammation and mitochondrial dysfunction is a key molecular target of POI ovarian lesions.

### 4.4 Gynecological malignancies

Gynecological malignancies such as ovarian cancer and endometrial cancer exhibit metabolic plasticity. The gut microbiota–mitochondria axis is an important factor in regulating tumor metabolism, and can affect the tumor microenvironment (TME) by regulating metabolic reprogramming, immune cell infiltration, and endocrine sensitivity (93).

#### 4.4.1 Clinical evidence and human observations in gynecological malignancies

Clinical cohort studies have found that specific gut microbiota composition characteristics are closely related to the progression of gynecological malignancies and the efficacy of chemotherapy. In the tumor microenvironment of patients with ovarian cancer and endometrial cancer, alterations in pro-inflammatory bacterial taxa, including *Fusobacterium nucleatum*, have been reported, which is associated with poor clinical prognosis and poor response to platinum-based chemotherapy (147, 148). Metabolomics analysis also showed that the patients' circulating BAs and estrogen metabolites changed significantly, reflecting the status of the intestinal estrobolome and correlating with systemic inflammatory indices (8, 148). However, a persistent translational challenge is distinguishing whether these microbial changes are primary drivers of tumorigenesis or secondary consequences of tumor-associated metabolic shifts and systemic anticancer therapies.

Regarding clinical translation, research primarily focuses on mitigating treatment toxicity and optimizing immunotherapy outcomes. A meta-analysis indicated that probiotic supplementation in postoperative endometrial cancer patients enhances microbial diversity and alleviates gastrointestinal symptoms, though without clear oncological benefit (149). Additionally, fecal microbiota transplantation (FMT) combined with immune checkpoint inhibitors has shown promising response rates in solid tumors (150). For ovarian cancer, a randomized trial of probiotic intervention in patients receiving platinum-based chemotherapy is currently underway (151). Despite these efforts, direct evidence linking gut-targeted interventions to the modulation of the tumor microenvironment, mitochondrial bioenergetics, or improved clinical prognosis in gynecological malignancies remains lacking (149, 152).

#### 4.4.2 Evidence from animal models of gynecological malignancies

Animal models of gynecological malignancies, including patient-derived xenografts and chemically induced tumor models, have provided mechanistic evidence supporting the involvement of the gut microbiota in shaping the tumor microenvironment. At the mechanistic level, intestinal metabolites such as SCFAs and secondary BAs act as systemic signaling molecules to regulate mitochondrial respiration of tumor cells. Altered bile acid signaling may also influence FXR/TGR5-mediated immune and metabolic regulation within the tumor microenvironment. Reduced SCFA availability may also impair HDAC-associated epigenetic regulation, thereby contributing to tumor-promoting metabolic and immune dysregulation within the tumor microenvironment (96). Endotoxemia caused by dysbiosis activates TLR4 signaling in the tumor microenvironment, thereby contributing to immunosuppressive cells and angiogenesis (153). At the same time, the gut microbiota–mitochondria axis can regulate the immune state of the tumor microenvironment; dysbiosis alters the mitochondrial function of tumor-infiltrating lymphocytes (TILs): insufficient intestinal SCFAs (especially butyrate) impair oxidative phosphorylation and memory formation in CD8+ T cells, leading to early T cell exhaustion and a decline in anti-tumor immune surveillance (154). Excessive production of mitochondrial ROS in the tumor microenvironment promotes tumor-associated macrophages (TAMs) to polarize towards the tumor-promoting M2 type, thus creating favorable conditions for tumor progression and metastasis (155). Certain bacteria, such as *F. nucleatum* have also been reported to enhance the metabolic plasticity of tumor cells, enabling them to adapt to the nutrient deficiency and hypoxic microenvironment common in gynecological malignancies (147). Experimental studies further suggested that dysregulated mitochondrial dynamics, particularly enhanced Drp1-mediated mitochondrial fission, may contribute to tumor proliferation and chemotherapy resistance, including reduced cisplatin sensitivity in gynecological malignancies (95, 156).

#### 4.4.3 Evidence from *in vitro* studies in gynecological malignancies

At the cellular level, the microbiota–mitochondria–apoptosis axis determines the survival and drug sensitivity of tumor cells. Estrobolome-mediated microbial metabolism regulates systemic estrogen levels through β-glucuronidase, up-regulates the glycolysis pathway of estrogen receptor-positive tumor cells, and strengthens mitochondrial fatty acid oxidation (8, 157). At the same time, mitochondrial stress is a core mediating factor of chemotherapy resistance. *In vitro* experiments show that microbial signals can alter the mitochondrial membrane potential, regulate cytochrome C release, and increase the apoptotic threshold (158, 159). Some microbial factors interfere with the quality control mechanism of mitophagy (PINK1/Parkin), potentially allowing tumor cells to evade mitochondrial-dependent apoptosis in the presence of taxane chemotherapy (160). The above research shows that targeting mitochondrial metabolic plasticity and microbiota-induced immunosuppression are important directions to overcome drug resistance in gynecological malignancies. At the molecular level, the interaction between mitochondrial stress and innate immune induction influences the efficacy of the tumor immune cycle. In theory, damaged mitochondria in gynecological tumor cells release mtDNA into the cytoplasm, which can activate the cGAS–STING pathway and recruit anti-tumor dendritic cells, but pathogenic bacteria such as *F. nucleatum* can inhibit this reaction and weaken the interferon-mediated immune killing effect (161, 162). The interaction of microbial signals and mitochondrial dysfunction may represent an important mechanism contributing to tumor immune escape.

## 5 Intervention and translational prospects

### 5.1 Microbiota intervention: probiotics, prebiotics, and fecal microbiota transplantation

**Figure 5:**
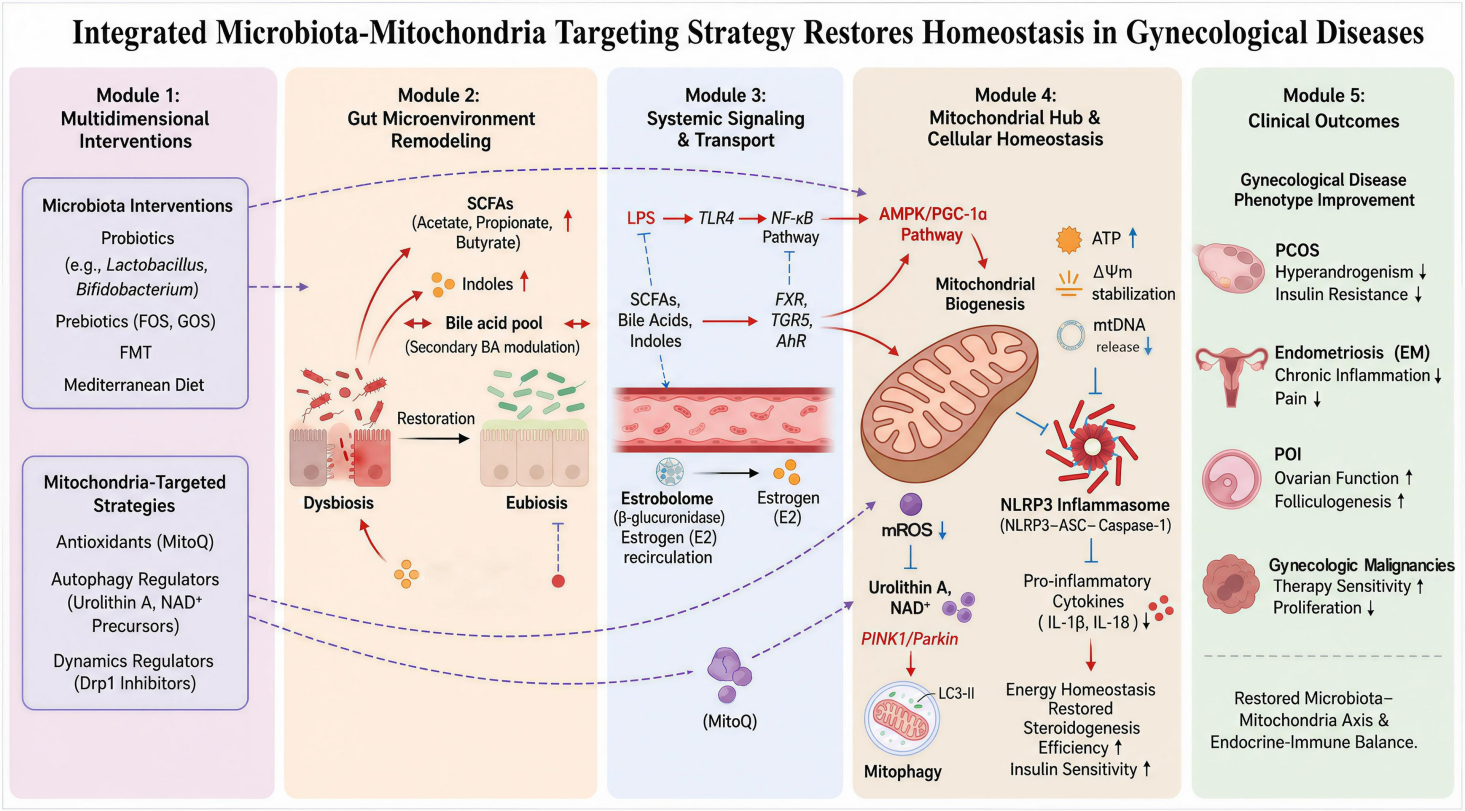
Integrated microbiota–mitochondria co-targeting strategy for gynecologic diseases. Module 1 outlines microbiota-directed interventions (diet, probiotics/prebiotics, and FMT) together with mitochondria-targeted approaches, including antioxidant, autophagy/mitophagy, and mitochondrial dynamics–related strategies. Module 2 depicts the restoration of microbial homeostasis (eubiosis) and metabolite outputs (SCFAs, bile acids, and indoles) alongside a reduced endotoxin (LPS) burden. Module 3 highlights key sensing and signaling pathways, including the inhibition of LPS–TLR4–NF-κB signaling, activation of FXR/TGR5 and AhR, and estrobolome-dependent estrogen recycling. Module 4 integrates these signals at the mitochondrial hub, demonstrating how AMPK–PGC-1α–associated mitochondrial biogenesis, stable mitochondrial homeostasis, and PINK1/Parkin-mediated mitophagy act collectively to limit mROS and mtDNA release, thereby dampening NLRP3 inflammasome activity. Module 5 summarizes the potential improvements in metabolic, endocrine, inflammatory, and therapeutic-response phenotypes across PCOS, endometriosis, POI, and gynecologic malignancies. Solid arrows indicate signaling or activation pathways, blue T-shaped lines indicate inhibitory effects, and dashed arrows represent intervention-associated modulation and delivery. FMT, fecal microbiota transplantation; FOS, fructooligosaccharides; GOS, galactooligosaccharides; SCFAs, short-chain fatty acids; FXR, farnesoid X receptor; TGR5, Takeda G protein-coupled receptor 5; AhR, aryl hydrocarbon receptor; LC3, microtubule-associated protein 1A/1B-light chain 3; mROS, mitochondrial reactive oxygen species; mtDNA, mitochondrial DNA; PCOS, polycystic ovary syndrome; POI, premature ovarian insufficiency.

Based on the framework of “metabolic input–immune amplification–oxidative stress/mitophagy–endocrine regulation” schematically summarized in Figure 5, intervention strategies targeting the microbiota are fundamental means to regulate the gut-ovarian axis, and the core translational goals are to restore microbial diversity, correct metabolite imbalance, and reduce the systemic endotoxin load.

#### 5.1.1 Probiotics and prebiotics

Probiotics, such as *Lactobacillus* spp., can regulate the function of the gut-ovarian axis by competitively inhibiting pathogenic bacteria and strengthening the integrity of the mucosal barrier (163). Preclinical studies have shown that this type of intervention can regulate the LPS–TLR4–NF-κB axis, reduce NLRP3 inflammasome activation, and support mitochondrial biogenesis (164). Prebiotics such as fructooligosaccharides and inulin can selectively promote the proliferation of SCFA-producing taxa (165). SCFAs can enhance antioxidant defense, promote PINK1/Parkin-mediated mitophagy, and reduce mtDNA cytoplasmic release (166). However, the intervention effects of probiotics and prebiotics are greatly influenced by strain specificity, host gut microbiota composition, and dosage. At present, the heterogeneity of clinical research is high, and the evidence is insufficient. It is necessary to carry out high-quality longitudinal clinical trials and clarify the applicable population and optimal preparation regimen to achieve clinical translation and application.

#### 5.1.2 Fecal microbiota transplantation (FMT)

FMT is a technique to quickly restore intestinal ecological balance and metabolic homeostasis by transplanting complex microbial communities (167). Theoretically, FMT can reconstruct the metabolic profile of SCFAs and BAs, relieve systemic inflammation, and reset the gut microbiota–mitochondria axis (168). Animal models and early population-based exploratory studies show that this technology can improve metabolic and inflammatory indices, but it is still in the experimental stage in the field of gynecological diseases (169). The standardization of donor screening, long-term safety, the risk of drug-resistant gene transfer, and the durability of the transplantation effect have not yet been resolved (170). Therefore, FMT can only be used in strictly monitored clinical trials or specific compassionate use scenarios that meet ethical and regulatory requirements.

### 5.2 Mitochondria-targeted strategies: antioxidants and autophagy regulators

Oxidative stress and impaired mitochondrial quality control are central links between gut microbiota dysbiosis and endocrine disorders, so therapeutic strategies targeting mitochondrial homeostasis have important research value, with the core being to alleviate oxidative damage and restore the dynamic balance of mitochondrial biogenesis and renewal.

#### 5.2.1 Antioxidants and metabolic modulators

MitoQ and other targeted mitochondrial antioxidants can selectively enrich in the mitochondrial matrix, scavenge mitochondrial ROS and maintain membrane potential. Preclinical studies of ovarian cancer and PCOS models show that MitoQ can alleviate lipid peroxidation and improve insulin signaling or chemotherapy-induced apoptosis (171, 172). Metabolic modulators such as NAD+ precursors can improve the efficiency of the tricarboxylic acid cycle, strengthen oxidative phosphorylation, and repair energy metabolism disorders (173, 174). However, the translational value of these drugs in the field of gynecology still needs to be verified, and the optimal dosage, tissue-specific delivery mode, and interaction with standard chemotherapy regimens need to be clarified before clinical application.

#### 5.2.2 Mitophagy and quality control regulator

Repairing mitochondrial quality control function, especially mitophagy and dynamic regulation, can reduce the release of DAMPs such as mtDNA, and is an important adjunctive treatment direction. Drugs such as urolithin A and spermidine have been shown in preclinical studies to promote PINK1/Parkin-mediated mitophagy, inhibit NLRP3 inflammasome assembly, and restore cellular homeostasis (90, 175). Regulating mitochondrial dynamics, for example by inhibiting excessive fission via Drp1, can promote mitochondrial fusion and improve energy metabolism efficiency in tumor cells or granulosa cells (95, 176). However, this type of drug is still in the research and development stage, and problems such as difficulty in systemic delivery and risk of off-target effects need to be handled cautiously.

### 5.3 Conceptual dual-target translational framework

The pathological cycle of gynecological diseases is complex, and it is difficult to completely block it with a single intervention method. Therefore, as schematically summarized in Figure 5, an integrated “dual-targeting” framework combining upstream microbiota regulation with downstream mitochondrial repair may represent a promising translational approach.

The core logic of this integrated approach is to block the innate immune amplification reaction at two levels: upstream microecological intervention (prebiotics, probiotics, microbiota-modulating diet) can optimize metabolic input, reduce systemic endotoxemia, and reduce the exogenous trigger signals for cell surface receptors such as TLR4 (177); Downstream targeted mitochondrial intervention can scavenge mitochondrial ROS, enhance PINK1/Parkin-mediated mitophagy, and prevent the release of DAMPs such as mtDNA (178).

From a mechanistic point of view, this dual blockade can break the activation mode of the NLRP3 inflammasome: upstream intervention weakens the LPS-induced NF-κB transcriptional initiation signal, and downstream intervention inhibits the activation signal caused by mitochondrial oxidative stress and structural collapse (179).

Applying this theoretical framework to translational research requires multi-dimensional evaluation rather than empirical treatment. Follow-up preclinical models and population-based exploratory research need to simultaneously monitor changes in intestinal microecology, systemic immune indicators, and easily accessible mitochondrial function indicators in peripheral blood mononuclear cells (180). The integrated model provides a hypothetical basis for follow-up studies and facilitates the understanding of the interactions among gut microbiota dysbiosis, cellular energy metabolism, and endocrine lesions.

### 5.4 Heterogeneity and precision translational strategies

The “microbiota-mitochondrial dual targeting framework” provides a feasible idea for blocking innate immune amplification reactions, but individual heterogeneity should be fully considered in clinical translation. Factors such as body mass index, insulin resistance, diet, region, and medication history of patients with gynecological diseases will affect the basal microecology and metabolic homeostasis (181), which is also the reason for the inconsistency of microbial signals in existing cohorts, and it also shows that a single fixed intervention program cannot be applied to all patients.

Therefore, the clinical translation of this framework needs to implement individualized and precise stratification strategies. There are differences in the basal microbial communities among hosts, and their responses to microecological regulators such as probiotics and prebiotics and to mitochondrial-targeted drugs show obvious individual differences (182).

To enhance the translational value of the dual-targeting strategy, future clinical trials need to shift from single biomarker monitoring to multi-omics-driven mechanistic phenotyping. For example, through integrative omics analysis, patients could be stratified into an endotoxin/inflammation-driven type and a mitochondrial intrinsic damage type, and matched with microbiota- or mitochondria-targeted intervention programs (183). Acknowledging and systematically studying individual heterogeneity will not reduce the mechanistic value of the framework, but will ensure that subsequent translational research is carried out precisely, safely, and individually.

## 6 Future perspectives

Building on the current understanding summarized above, future research should focus on advancing clinical translation and therapeutic strategies in this field. In the future, research in this field can focus on analyzing the precise molecular mechanisms of the gut microbiota–mitochondria axis interaction, and promote related research from correlative description to causal exploration and high-resolution spatial localization studies.

### 6.1 Exploring causality: germ-free models and 3D organoids

At present, most microbiota research in gynecological diseases is based on correlative analyses, such as changes in fecal microbial taxonomy, and it is necessary to explore causality with the help of advanced biological models. In the future, we can transplant patients' fecal microbiota into germ-free (GF) mouse models and observe whether mitochondrial dysfunction and disease phenotype are induced after colonization (86). At the same time, the construction of an *in vitro* system for co-culture of 3D organoids and immune cells from gynecological diseases is also worthy of further study (184). This kind of microphysiological system can verify whether specific strains or metabolites directly influence mitochondrial damage and activate the NLRP3 inflammasome, and offering a more robust platform for mechanistic studies.

### 6.2 Advancing spatial omics: decoding *in situ* immune-mitochondrial crosstalk

Traditional bulk sequencing technologies lose key information about tissue structure and the spatial microenvironment, and gynecological diseases such as the ovarian cancer TME and endometriosis lesions have strong spatial heterogeneity (185). Therefore, future research needs to analyze the interaction between *in situ* immunity and mitochondria with the help of spatial transcriptomics and spatial metabolomics (186). This kind of *in situ* study is at the frontier of mucosal immunology, and can precisely locate the distribution of microbial DNA and metabolites in tissues, clarify their spatial interactions with local macrophages, and simultaneously capture changes in host cell mitochondrial stress genes (187). An integrated cross-scale regulatory map of “microbiota–metabolite–mitochondrial state–tissue microenvironment” can be constructed by combining spatial omics and single-cell microenvironment technologies.

### 6.3 Improving methodological standardization in microbiota-immune research

The core bottleneck of current research, which is also the focus of peer review, is that the heterogeneity of methodologies is too high, leading to an inability to replicate research results. Factors such as diet, body mass index, and recent medication history can interfere with bioinformatics analysis results. Therefore, it is necessary to formulate and strictly implement standardized research norms in this field as soon as possible (188). In the future, a field consensus needs to clarify and unify operating procedures, standardize DNA extraction kits and analysis pipelines, and reduce batch effects (189). At the same time, it is necessary to clearly distinguish between fecal-resident microbiota and tissue-resident mucosal microbiota, as their immunomodulatory effects on mitochondria are significantly different (190). Adopting causal inference algorithms, controlling for multi-cohort confounding factors, and pre-registering study protocols could enhance the rigor of microbiota-immune research and facilitate the standardized development of the field (191).

## 7 Conclusion

In summary, this study puts forward a new closed-loop model of “microbiota-immunoamplification-mitochondria-endocrine”, which changes the research paradigm of gynecological pathophysiology from a single-organ perspective to a systemic multi-system interactive perspective. It clarifies that gut microbiota dysbiosis is considered a key contributing factor, and that immune cells, especially macrophages, act as amplification mediators to induce mitochondrial dysfunction, which in turn leads to endocrine disorders and promotes the occurrence and development of diseases such as endometriosis, polycystic ovary syndrome, and gynecological malignancies. Interventions targeting this regulator*y* axis, such as probiotics and mitochondria-targeted drugs, have good translational prospects, but the first task at present is to rigorously elucidate the relevant pathway mechanisms through spatial multi-omics and standardized causal experimental models. Analyzing the complex interaction between microbiota and mitochondria can provide new theoretical support for the research and development of individualized and precise diagnostic and therapeutic technologies for female reproductive system diseases.

## References

[1] Wang M Zheng L-W Ma S Zhao D-H Xu Y. The gut microbiota: emerging biomarkers and potential treatments for infertility-related diseases. Front Cell Infect Microbiol. (2024) 14:1450310. 10.3389/fcimb.2024.1450310 39391885 PMC11464459

[2] Zhou Z Feng Y Xie L Ma S Cai Z Ma Y. Alterations in gut and genital microbiota associated with gynecological diseases: a systematic review and meta-analysis. Reprod Biol Endocrinol. (2024) 22:13. 10.1186/s12958-024-01184-z 38238814 PMC10795389

[3] Zachos KA Gamboa JA Dewji AS Lee J Brijbassi S Andreazza AC. The interplay between mitochondria, the gut microbiome and metabolites and their therapeutic potential in primary mitochondrial disease. Front Pharmacol. (2024) 15:1428242. 10.3389/fphar.2024.1428242 39119601 PMC11306032

[4] Borbolis F Mytilinaiou E Palikaras K. The crosstalk between microbiome and mitochondrial homeostasis in neurodegeneration. Cells. (2023) 12:429. 10.3390/cells12030429 36766772 PMC9913973

[5] Qi X Yun C Pang Y Qiao J. The impact of the gut microbiota on the reproductive and metabolic endocrine system. Gut Microbes. (2021) 13:1894070. 10.1080/19490976.2021.1894070 33722164 PMC7971312

[6] Zhang M Hu R Huang Y Zhou F Li F Liu Z. Present and future: crosstalks between polycystic ovary syndrome and gut metabolites relating to gut microbiota. Front Endocrinol. (2022) 13:933110. 10.3389/fendo.2022.933110 PMC9343597 35928893

[7] Kumari N Kumari R Dua A Singh M Kumar R Singh P. From gut to hormones: unraveling the role of gut Microbiota in (phyto)Estrogen modulation in health and disease. Molecular Nutrition Food Res. (2024) 68:2300688. 10.1002/mnfr.202300688 38342595

[8] Hu S Ding Q Zhang W Kang M Ma J Zhao L. Gut microbial beta-glucuronidase: a vital regulator in female estrogen metabolism. Gut Microbes. (2023) 15:2236749. 10.1080/19490976.2023.2236749 37559394 PMC10416750

[9] Weber-Stiehl S Järke L Castrillón-Betancur JC Gilbert F Sommer F. Mitochondrial function and microbial metabolites as central regulators of intestinal immune responses and cancer. Front Microbiol. (2022) 13:919424. 10.3389/fmicb.2022.919424 35847099 PMC9277123

[10] Ballard JWO Towarnicki SG. Mitochondria, the gut microbiome and ROS. Cell Signal. (2020) 75:109737. 10.1016/j.cellsig.2020.109737 32810578

[11] Urbauer E Aguanno D Mindermann N Omer H Metwaly A Krammel T. Mitochondrial perturbation in the intestine causes microbiota-dependent injury and gene signatures discriminative of inflammatory disease. Cell Host Microbe. (2024) 32:1347–1364.e10. 10.1016/j.chom.2024.06.013 39013472

[12] Zhang Q Xing W Wang Q Tang Z Wang Y Gao W. Gut microbiota–mitochondrial inter-talk in non-alcoholic fatty liver disease. Front Nutr. (2022) 9:934113. 10.3389/fnut.2022.934113 36204383 PMC9530335

[13] Ju T Zhang Y Liu L Zhao X Li X Liu C. The role of gut microbiota–mitochondria crosstalk in neurodegeneration: underlying mechanisms and potential therapies. Neural Regen Res. (2026) 21:2238. 10.4103/NRR.NRR-D-24-01419 40314217 PMC13211786

[14] Senthilkumar H Arumugam M. Gut microbiota: a hidden player in polycystic ovary syndrome. J Transl Med. (2025) 23:443. 10.1186/s12967-025-06315-7 40234859 PMC11998441

[15] Mei Y Li W Wang B Chen Z Wu X Lin Y. Gut microbiota: an emerging target connecting polycystic ovarian syndrome and insulin resistance. Front Cell Infect Microbiol. (2025) 15:1508893. 10.3389/fcimb.2025.1508893 40134784 PMC11933006

[16] Baușic AIG Scurtu F Manu A Matasariu DR Brătilă E. Gut Microbiota dysbiosis in endometriosis: a potential link to inflammation and disease progression. IJMS. (2025) 26:5144. 10.3390/ijms26115144 40507956 PMC12153989

[17] Tang F Deng M Xu C Yang R Ji X Hao M. Unraveling the microbial puzzle: exploring the intricate role of gut microbiota in endometriosis pathogenesis. Front Cell Infect Microbiol. (2024) 14:1328419. 10.3389/fcimb.2024.1328419 38435309 PMC10904627

[18] Huang F Cao Y Liang J Tang R Wu S Zhang P. The influence of the gut microbiome on ovarian aging. Gut Microbes. (2024) 16:2295394. 10.1080/19490976.2023.2295394 38170622 PMC10766396

[19] Peng Y Wang Y Hu J Wang Z Liu Y Ding Z. Trimethylamine N-oxide (TMAO) treatment triggers premature ovarian insufficiency (POI) via the activation of mitochondrial pathway apoptosis in granulosa cells. Free Radic Biol Med. (2025) 232:214–30. 10.1016/j.freeradbiomed.2025.03.007 40054636

[20] Ma J Yao Z Ma L Zhu Q Zhang J Li L. Glucose metabolism reprogramming in gynecologic malignant tumors. J Cancer. (2024) 15:2627–45. 10.7150/jca.91131 38577616 PMC10988310

[21] Chartoumpekis DV Zaravinos A Apidianakis Y Lagoumintzis G. Editorial: microbiota and mitochondria: impact on cell signaling, physiology, and disease. Front Microbiol. (2022) 13:1056499. 10.3389/fmicb.2022.1056499 36329843 PMC9623293

[22] Zhu Y Li Y Zhang Q Song Y Wang L Zhu Z. Interactions between intestinal microbiota and neural mitochondria: a new perspective on communicating pathway from gut to brain. Front Microbiol. (2022) 13:798917. 10.3389/fmicb.2022.798917 35283843 PMC8908256

[23] Wang L He L Xu L Li S. Short-chain fatty acids: bridges between diet, gut microbiota, and health. J of Gastro and Hepatol. (2024) 39:1728–36. 10.1111/jgh.16619 38780349

[24] Masse KE Lu VB. Short-chain fatty acids, secondary bile acids and indoles: gut microbial metabolites with effects on enteroendocrine cell function and their potential as therapies for metabolic disease. Front Endocrinol. (2023) 14:1169624. 10.3389/fendo.2023.1169624 PMC10407565 37560311

[25] Vezza T Abad-Jiménez Z Marti-Cabrera M Rocha M Víctor VM. Microbiota-mitochondria inter-talk: a potential therapeutic strategy in obesity and type 2 diabetes. Antioxidants. (2020) 9:848. 10.3390/antiox9090848 32927712 PMC7554719

[26] Zhang Y Zhang J Duan L. The role of microbiota-mitochondria crosstalk in pathogenesis and therapy of intestinal diseases. Pharmacol Res. (2022) 186:106530. 10.1016/j.phrs.2022.106530 36349593

[27] Shen Y Fan N Ma S Cheng X Yang X Wang G. Gut microbiota dysbiosis: pathogenesis, diseases, prevention, and therapy. MedComm. (2025) 6:e70168. 10.1002/mco2.70168 40255918 PMC12006732

[28] Frampton J Murphy KG Frost G Chambers ES. Short-chain fatty acids as potential regulators of skeletal muscle metabolism and function. Nat Metab. (2020) 2:840–8. 10.1038/s42255-020-0188-7 32694821

[29] Jin C Chen H Xie L Zhou Y Liu L Wu J. GPCRs involved in metabolic diseases: pharmacotherapeutic development updates. Acta Pharmacol Sin. (2024) 45:1321–36. 10.1038/s41401-023-01215-2 38326623 PMC11192902

[30] Perino A Demagny H Velazquez-Villegas L Schoonjans K. Molecular physiology of bile acid signaling in health, disease, and aging. Physiol Rev. (2021) 101:683–731. 10.1152/physrev.00049.2019 32790577

[31] Sui Y Wu J Chen J. The role of gut microbial β-glucuronidase in estrogen reactivation and breast cancer. Front Cell Dev Biol. (2021) 9:631552. 10.3389/fcell.2021.631552 34458248 PMC8388929

[32] Platten M Nollen EAA Röhrig UF Fallarino F Opitz CA. Tryptophan metabolism as a common therapeutic target in cancer, neurodegeneration and beyond. Nat Rev Drug Discov. (2019) 18:379–401. 10.1038/s41573-019-0016-5 30760888

[33] Chen J Jia S Xue X Guo C Dong K. Gut microbiota: a novel target for exercise-mediated regulation of NLRP3 inflammasome activation. Front Microbiol. (2025) 15:1476908. 10.3389/fmicb.2024.1476908 39834360 PMC11743191

[34] Pan H Jian Y Wang F Yu S Guo J Kan J. NLRP3 And gut microbiota homeostasis: progress in research. Cells. (2022) 11:3758. 10.3390/cells11233758 36497018 PMC9739202

[35] Manshouri S Seif F Kamali M Bahar MA Mashayekh A Molatefi R. The interaction of inflammasomes and gut microbiota: novel therapeutic insights. Cell Commun Signal. (2024) 22:209. 10.1186/s12964-024-01504-1 38566180 PMC10986108

[36] Billingham LK Stoolman JS Vasan K Rodriguez AE Poor TA Szibor M. Mitochondrial electron transport chain is necessary for NLRP3 inflammasome activation. Nat Immunol. (2022) 23:692–704. 10.1038/s41590-022-01185-3 35484407 PMC9098388

[37] Riley JS Tait SW. Mitochondrial DNA in inflammation and immunity. EMBO Rep. (2020) 21:EMBR201949799. 10.15252/embr.201949799 PMC7132203 32202065

[38] Yang D Wang Z Chen Y Guo Q Dong Y. Interactions between gut microbes and NLRP3 inflammasome in the gut-brain axis. Comput Struct Biotechnol J. (2023) 21:2215–27. 10.1016/j.csbj.2023.03.017 37035548 PMC10074411

[39] Zheng D Liwinski T Elinav E. Inflammasome activation and regulation: toward a better understanding of complex mechanisms. Cell Discov. (2020) 6:36. 10.1038/s41421-020-0167-x 32550001 PMC7280307

[40] Salliss ME Farland LV Mahnert ND Herbst-Kralovetz MM. The role of gut and genital microbiota and the estrobolome in endometriosis, infertility and chronic pelvic pain. Hum Reprod Update. (2022) 28:92–131. 10.1093/humupd/dmab035 34718567

[41] Guo X Xu X Li T Yu Q Wang J Chen Y. NLRP3 Inflammasome activation of mast cells by estrogen via the nuclear-initiated signaling pathway contributes to the development of endometriosis. Front Immunol. (2021) 12:749979. 10.3389/fimmu.2021.749979 34630429 PMC8494307

[42] Li W Gui Y Guo C Huang Y Liu Y Yu X. Molecular mechanisms of mitochondrial quality control. Transl Neurodegener. (2025) 14:45. 10.1186/s40035-025-00505-5 40887660 PMC12400733

[43] Kassan M Kwon Y Munkhsaikhan U Sahyoun AM Ishrat T Galán M. Protective role of short-chain fatty acids against ang- II-induced mitochondrial dysfunction in brain endothelial cells: a potential role of heme oxygenase 2. Antioxidants. (2023) 12:160. 10.3390/antiox12010160 36671022 PMC9854784

[44] Zeng N Wu F Lu J Li X Lin S Zhou L. High-fat diet impairs gut barrier through intestinal microbiota-derived reactive oxygen species. Sci China Life Sci. (2024) 67:879–91. 10.1007/s11427-022-2283-4 37202543

[45] Xie X Huang C. Role of the gut–muscle axis in mitochondrial function of ageing muscle under different exercise modes. Ageing Res Rev. (2024) 98:102316. 10.1016/j.arr.2024.102316 38703951

[46] Green A Hossain T Eckmann DM. Mitochondrial dynamics involves molecular and mechanical events in motility, fusion and fission. Front Cell Dev Biol. (2022) 10:1010232. 10.3389/fcell.2022.1010232 36340034 PMC9626967

[47] Mishra SR Mahapatra KK Behera BP Patra S Bhol CS Panigrahi DP. Mitochondrial dysfunction as a driver of NLRP3 inflammasome activation and its modulation through mitophagy for potential therapeutics. Int J Biochem Cell Biol. (2021) 136:106013. 10.1016/j.biocel.2021.106013 34022434

[48] Song Y Zhou Y Zhou X. The role of mitophagy in innate immune responses triggered by mitochondrial stress. Cell Commun Signal. (2020) 18:186. 10.1186/s12964-020-00659-x 33239048 PMC7687798

[49] Zhang W Kong L Zhong Z Lin L Li J Zheng G. Short chain fatty acids increase fat oxidation and promote browning through β3-adrenergic receptor/AMP-activated protein kinase *α* signaling pathway in 3T3-L1 adipocytes. J Funct Foods. (2023) 103:105488. 10.1016/j.jff.2023.105488

[50] Aqeel A Akram A Ali M Iqbal M Aslam M Rukhma Mechanistic insights into impaired β-oxidation and its role in mitochondrial dysfunction: a comprehensive review. Diabetes Res Clin Pract. (2025) 223:112129. 10.1016/j.diabres.2025.112129 40132731

[51] Chen J Liu B Yao X Yang X Sun J Yi J. AMPK/SIRT1/PGC-1α signaling pathway: molecular mechanisms and targeted strategies from energy homeostasis regulation to disease therapy. CNS Neurosci Ther. (2025) 31:e70657. 10.1111/cns.70657 41268687 PMC12635776

[52] Zangerolamo L Carvalho M Barbosa HCL. The critical role of the bile acid receptor TGR5 in energy homeostasis: insights into physiology and therapeutic potential. Int J Mol Sci. (2025) 26:6547. 10.3390/ijms26146547 40724796 PMC12294878

[53] Yan W Zhang K Guo J Xu L. Bile acid-mediated gut-liver axis crosstalk: the role of nuclear receptor signaling in dynamic regulation of inflammatory networks. Front Immunol. (2025) 16:1595486. 10.3389/fimmu.2025.1595486 40458398 PMC12127205

[54] Chiang JYL Ferrell JM. Bile acid receptors FXR and TGR5 signaling in fatty liver diseases and therapy. Am J Physiol Gastrointestinal Liver Physiol. (2020) 318:G554–73. 10.1152/ajpgi.00223.2019 PMC7099488 31984784

[55] Siemers KM Klein AK Baack ML. Mitochondrial dysfunction in PCOS: insights into reproductive organ pathophysiology. Int J Mol Sci. (2023) 24:13123. 10.3390/ijms241713123 37685928 PMC10488260

[56] Tang B Tang L Li S Liu S He J Li P. Gut microbiota alters host bile acid metabolism to contribute to intrahepatic cholestasis of pregnancy. Nat Commun. (2023) 14:1305. 10.1038/s41467-023-36981-4 36894566 PMC9998625

[57] Li Q de Oliveira Formiga R Puchois V Creusot L Ahmad AH Amouyal S. Microbial metabolite indole-3-propionic acid drives mitochondrial respiration in CD4+ T cells to confer protection against intestinal inflammation. Nat Metab. (2025) 7:2510–30. 10.1038/s42255-025-01396-6 41120706 PMC12727523

[58] Lu Z Zhang C Zhang J Su W Wang G Wang Z. The kynurenine pathway and indole pathway in tryptophan metabolism influence tumor progression. Cancer Med. (2025) 14:e70703. 10.1002/cam4.70703 40103267 PMC11919716

[59] Savitz J. The kynurenine pathway: a finger in every pie. Mol Psychiatry. (2020) 25:131–47. 10.1038/s41380-019-0414-4 30980044 PMC6790159

[60] Higgins B Simitsidellis I Zheng X Collins F Homer NZ Denham SG. Kynurenine monooxygenase blockade reduces endometriosis-like lesions, improves visceral hyperalgesia, and rescues mice from a negative behavioural phenotype in experimental endometriosis. eLife. (2024) 13:99226. 10.7554/eLife.99226.2

[61] Wang S Mu L Zhang C Long X Zhang Y Li R. Abnormal activation of tryptophan-kynurenine pathway in women with polycystic ovary syndrome. Front Endocrinol. (2022) 13:877807. 10.3389/fendo.2022.877807 PMC9199373 35721725

[62] Di Vincenzo F Del Gaudio A Petito V Lopetuso LR Scaldaferri F. Gut microbiota, intestinal permeability, and systemic inflammation: a narrative review. Intern Emerg Med. (2024) 19:275–93. 10.1007/s11739-023-03374-w 37505311 PMC10954893

[63] Samanta S Mukherjee G Chattopadhyay S Bishayi B. Concomitant inhibition of TLR4 and NLRP3 inflammasome in macrophages regulates lipopolysaccharide induced inflammation. The Microbe. (2025) 8:100414. 10.1016/j.microb.2025.100414

[64] Liu J Wang M Zhao Y. The regulatory network of transcription factors in macrophage polarization. Immunotargets Ther. (2025) 14:555–75. 10.2147/ITT.S494550 40496968 PMC12151090

[65] Kim J Kim H-S Chung JH. Molecular mechanisms of mitochondrial DNA release and activation of the cGAS-STING pathway. Exp Mol Med. (2023) 55:510–9. 10.1038/s12276-023-00965-7 36964253 PMC10037406

[66] Li R Liu H Liu Y. The cGAS-STING pathway and female reproductive system diseases. Front Immunol. (2024) 15:1447719. 10.3389/fimmu.2024.1447719 39445027 PMC11496054

[67] Chen X Zhu Y Xia L Su S Fan S Lu Y. Glutamine limits NLRP3 inflammasome activation and pyroptosis in macrophages by sustaining the IRG1/itaconate axis. FEBS J. (2025) 292:6016–34. 10.1111/febs.70119 40296302

[68] Hu Y Yang Y Li Y Zhang Q Zhang W Jia J. Th17/treg imbalance in inflammatory bowel disease: immunological mechanisms and microbiota-driven regulation. Front Immunol. (2025) 16:1651063. 10.3389/fimmu.2025.1651063 41132656 PMC12540096

[69] Liu X Chen J Yue S Zhang C Song J Liang H. NLRP3-mediated IL-1β in regulating the imbalance between Th17 and Treg in experimental autoimmune prostatitis. Sci Rep. (2024) 14:18829. 10.1038/s41598-024-69512-2 39138267 PMC11322183

[70] Dai Y Chen Y. Targeting persistently activated inflammatory microenvironment to promote chronic wound healing. Front Immunol. (2025) 16:1708358. 10.3389/fimmu.2025.1708358 41479883 PMC12753479

[71] Zhang D Zhang B. cGAS/STING signaling pathway in gynecological malignancies: from molecular mechanisms to therapeutic values. Front Immunol. (2025) 16:1525736. 10.3389/fimmu.2025.1525736 39949780 PMC11821648

[72] Mostafavi Abdolmaleky H Zhou J-R. Gut microbiota dysbiosis, oxidative stress, inflammation, and epigenetic alterations in metabolic diseases. Antioxidants. (2024) 13:985. 10.3390/antiox13080985 39199231 PMC11351922

[73] Sun Y Wang X Li L Zhong C Zhang Y Yang X. The role of gut microbiota in intestinal disease: from an oxidative stress perspective. Front Microbiol. (2024) 15:1328324. 10.3389/fmicb.2024.1328324 38419631 PMC10899708

[74] Narendra DP Youle RJ. The role of PINK1–Parkin in mitochondrial quality control. Nat Cell Biol. (2024) 26:1639–51. 10.1038/s41556-024-01513-9 39358449

[75] Thayer JA Petersen JD Huang X Gruel Budet LM Hawrot J Ramos DM. A unified mechanism for mitochondrial damage sensing in PINK1-Parkin–mediated mitophagy. EMBO J. (2026) 45:64–105. 10.1038/s44318-025-00604-z 41266657 PMC12759083

[76] Okatsu K Fukai S. Ubiquitin signaling in PINK1/Parkin-dependent mitophagy. J Biochem. (2026) 179:145–54. 10.1093/jb/mvaf079 41368810

[77] Sun S Hou H Ma G Ma Q Li N Zhang L. The interaction between E3 ubiquitin ligase Parkin and mitophagy receptor PHB2 links inner mitochondrial membrane ubiquitination to efficient mitophagy. J Biol Chem. (2022) 298:102704. 10.1016/j.jbc.2022.102704 36379251 PMC9763867

[78] Lechado-Terradas A Schepers S Zittlau KI Sharma K Ok O Fitzgerald JC. Parkin-dependent mitophagy occurs via proteasome-dependent steps sequentially targeting separate mitochondrial sub-compartments for autophagy. Autophagy Rep. (2022) 1:576–602. 10.1080/27694127.2022.2143214 40396016 PMC11864704

[79] Marcuzzo MB de Andrade Silveira J Pinheiro CV da Rosa JS Zemniaçak AB Brondani M. Intracerebral administration of hydrogen sulfide impairs bioenergetics, redox status and mitochondrial quality control in rat striatum. Neurotox Res. (2025) 43:35. 10.1007/s12640-025-00758-y 40974451

[80] Nicese MN Bijkerk R Van Zonneveld AJ Van den Berg BM Rotmans JI. Sodium butyrate as key regulator of mitochondrial function and barrier integrity of human glomerular endothelial cells. Int J Mol Sci. (2023) 24:13090. 10.3390/ijms241713090 37685905 PMC10487840

[81] Gąssowska-Dobrowolska M Olech-Kochańczyk G Culmsee C Adamczyk A. Novel insights into parkin–mediated mitochondrial dysfunction and “mito-inflammation” in α-synuclein toxicity. The role of the cGAS–STING signalling pathway. J Inflamm Res. (2024) 17:4549–74. 10.2147/JIR.S468609 39011416 PMC11249072

[82] Jiménez-Loygorri JI Boya P. Aging STINGs: mitophagy at the crossroads of neuroinflammation. Autophagy. (2024) 20:1684–6. 10.1080/15548627.2024.2322421 38411192 PMC11210893

[83] Tang Y Yang J Hang F Huang H Jiang L. Unraveling the relationship between gut microbiota and site-specific endometriosis: a Mendelian randomization analysis. Front Microbiol. (2024) 15:1363080. 10.3389/fmicb.2024.1363080 39027094 PMC11254793

[84] Li P Shuai P Shen S Zheng H Sun P Zhang R. Perturbations in gut microbiota composition in patients with polycystic ovary syndrome: a systematic review and meta-analysis. BMC Med. (2023) 21:302. 10.1186/s12916-023-02975-8 37559119 PMC10413517

[85] Li M Chang Q Luo Y Pan J Hu Y Liu B. The gut microbial composition in polycystic ovary syndrome with hyperandrogenemia and its association with steroid hormones. Front Cell Dev Biol. (2024) 12:1384233. 10.3389/fcell.2024.1384233 38872933 PMC11169812

[86] Huang F Deng Y Zhou M Tang R Zhang P Chen R. Fecal microbiota transplantation from patients with polycystic ovary syndrome induces metabolic disorders and ovarian dysfunction in germ-free mice. BMC Microbiol. (2024) 24:364. 10.1186/s12866-024-03513-z 39333864 PMC11437718

[87] Hanna A Abbas H Yassine F AlBush A Bilen M. Systematic review of gut microbiota composition, metabolic alterations, and the effects of treatments on PCOS and gut microbiota across human and animal studies. Front Microbiol. (2025) 16:1549499. 10.3389/fmicb.2025.1549499 40438215 PMC12116390

[88] Wu J Zhuo Y Liu Y Chen Y Ning Y Yao J. Association between premature ovarian insufficiency and gut microbiota. BMC Pregnancy Childbirth. (2021) 21:418. 10.1186/s12884-021-03855-w 34090383 PMC8180047

[89] Wang J Luo R Zhao X Xia D Liu Y Shen T. Association between gut microbiota and primary ovarian insufficiency: a bidirectional two-sample Mendelian randomization study. Front Endocrinol. (2023) 14:1183219. 10.3389/fendo.2023.1183219 PMC10324962 37424857

[90] Ju W Yan B Li D Lian F Xiang S. Mitochondria-driven inflammation: a new frontier in ovarian ageing. J Transl Med. (2025) 23:1005. 10.1186/s12967-025-06966-6 40993676 PMC12462319

[91] Liu Z Wang M Lei Y Xu K Fan L. Gut microbiota: emerging biomarkers and potential therapeutics for premature ovarian failure. Front Microbiol. (2025) 16:1606001. 10.3389/fmicb.2025.1606001 40735614 PMC12303985

[92] Ma Y Chen Y Yuan X Li T Luo H Gu Y. The inflammatory clock: how cGAS-STING ticks in the aging ovary. Front Cell Dev Biol. (2026) 14:1771546. 10.3389/fcell.2026.1771546 41816108 PMC12972751

[93] Han M Wang N Han W Ban M Sun T Xu J. Gut microbes in gynecologic cancers: causes or biomarkers and therapeutic potential. Front Oncol. (2022) 12:902695. 10.3389/fonc.2022.902695 35912194 PMC9326394

[94] Ghosh A Jaaback K Boulton A Wong-Brown M Raymond S Dutta P. Fusobacterium nucleatum: an overview of evidence, demi-decadal trends, and its role in adverse pregnancy outcomes and Various gynecological diseases, including cancers. Cells. (2024) 13:717. 10.3390/cells13080717 38667331 PMC11049087

[95] Javed Z Shin DH Pan W White SR Elhaw AT Kim YS. Drp1 splice variants regulate ovarian cancer mitochondrial dynamics and tumor progression. EMBO Rep. (2024) 25:4281–310. 10.1038/s44319-024-00232-4 39191946 PMC11467262

[96] Chen H Lou G Meng F Zhang Y Kuang H Yang D. Critical role of reproductive tract microbiota and derived metabolites in inflammation, tumor immunity, and tumorigenesis of gynecological cancers: a narrative review. Front Immunol. (2026) 17:1734792. 10.3389/fimmu.2026.1734792 41859112 PMC12996071

[97] Tierney BT Tan Y Yang Z Shui B Walker MJ Kent BM. Systematically assessing microbiome–disease associations identifies drivers of inconsistency in metagenomic research. PLoS Biol. (2022) 20:e3001556. 10.1371/journal.pbio.3001556 35235560 PMC8890741

[98] Tegegne HA Savidge TC. Gut microbiome metagenomics in clinical practice: bridging the gap between research and precision medicine. Gut Microbes. (2025) 17:2569739. 10.1080/19490976.2025.2569739 41137523 PMC12562794

[99] Dilliyappan S Kumar AS Venkatesalu S Palaniyandi T Baskar G Sivaji A. Polycystic ovary syndrome: recent research and therapeutic advancements. Life Sci. (2024) 359:123221. 10.1016/j.lfs.2024.123221 39521272

[100] Yang Y Cheng J Liu C Zhang X Ma N Zhou Z. Gut microbiota in women with polycystic ovary syndrome: an individual based analysis of publicly available data. eClinicalMed. (2024) 77:102884. 10.1016/j.eclinm.2024.102884 PMC11513668 39469535

[101] Yu Z Qin E Cheng S Yang H Liu R Xu T. Gut microbiome in PCOS associates to serum metabolomics: a cross-sectional study. Sci Rep. (2022) 12:22184. 10.1038/s41598-022-25041-4 36564416 PMC9789036

[102] Ding Y Jiang Y Zhu M Zhu Q He Y Lu Y. Follicular fluid lipidomic profiling reveals potential biomarkers of polycystic ovary syndrome: a pilot study. Front Endocrinol. (2022) 13:960274. 10.3389/fendo.2022.960274 PMC9513192 36176459

[103] Martinez Guevara D Vidal Cañas S Palacios I Gómez A Estrada M Gallego J. Effectiveness of probiotics, prebiotics, and synbiotics in managing insulin resistance and hormonal imbalance in women with polycystic ovary syndrome (PCOS): a systematic review of randomized clinical trials. Nutrients. (2024) 16:3916. 10.3390/nu16223916 39599701 PMC11597640

[104] Li J Qiao J Li Y Qin G Xu Y Lao K. Metabolic disorders in polycystic ovary syndrome: from gut microbiota biodiversity to clinical intervention. Front Endocrinol. (2025) 16:1526468. 10.3389/fendo.2025.1526468 PMC12066289 40357203

[105] Chudzicka-Strugała I Kubiak A Banaszewska B Wysocka E Zwozdziak B Siakowska M. Six-month randomized, placebo controlled trial of synbiotic supplementation in women with polycystic ovary syndrome undergoing lifestyle modifications. Arch Gynecol Obstet. (2025) 311:499–506. 10.1007/s00404-024-07833-3 39636391 PMC11890239

[106] Scannell N Mantzioris E Cowan S Moran L Villani A. A pilot randomized control trial evaluating the feasibility of a 12-week Mediterranean diet intervention without caloric restriction in women with polycystic ovary syndrome. J Clin Med. (2025) 14:5842. 10.3390/jcm14165842 40869668 PMC12386805

[107] Borzan V Riedl R Obermayer-Pietsch B. Probiotic vs. Placebo and metformin: probiotic dietary intervention in polycystic ovary syndrome—a randomized controlled trial. BMC Endocr Disord. (2023) 23:82. 10.1186/s12902-023-01294-6 37062834 PMC10106320

[108] Luo M Chen Y Pan X Chen H Fan L Wen Y. E. coli Nissle 1917 ameliorates mitochondrial injury of granulosa cells in polycystic ovary syndrome through promoting gut immune factor IL-22 via gut microbiota and microbial metabolism. Front Immunol. (2023) 14:1137089. 10.3389/fimmu.2023.1137089 37275915 PMC10235540

[109] Zhu S Chen H He B Zhang Y Li P Kuang J. Gut microbiota dysbiosis in polycystic ovary syndrome: focus on diet, probiotics, and traditional Chinese medicine. Front Microbiol. (2025) 16:1659783. 10.3389/fmicb.2025.1659783 41377049 PMC12685912

[110] Li X Cui Y Zhang C Zang W Cheng Y Yang C. Treatment of *Qin Gui Wan* (QGW) in PCOS abnormal oocytes development via AMPK/PGC-1ɑ pathway. J Ethnopharmacol. (2025) 342:119434. 10.1016/j.jep.2025.119434 39894417

[111] Zhao H Chen R Zheng D Xiong F Jia F Liu J. Modified banxia xiexin decoction ameliorates polycystic ovarian syndrome with insulin resistance by regulating intestinal microbiota. Front Cell Infect Microbiol. (2022) 12:854796. 10.3389/fcimb.2022.854796 35619648 PMC9127304

[112] Kobayashi H Shigetomi H Nishio M Umetani M Imanaka S Hashimoto H. Molecular mechanisms underlying reproductive dysfunction in polycystic ovary syndrome. Reprod Sci. (2026). 10.1007/s43032-026-02097-5 42010141

[113] Mobeen A Joshi S Fatima F Bhargav A Arif Y Faruq M. NF-κB signaling is the major inflammatory pathway for inducing insulin resistance. 3 Biotech. (2025) 15:47. 10.1007/s13205-024-04202-4 39845928 PMC11747027

[114] Wang Y Yang Q Wang H Zhu J Cong L Li H. NAD+ deficiency and mitochondrial dysfunction in granulosa cells of women with polycystic ovary syndrome‡. Biol Reprod. (2021) 105:371–80. 10.1093/biolre/ioab078 34056649

[115] Liu X Liu M Wang J Yang J Zhou X Liu C. Biomarker identification and mechanism of polycystic ovary syndrome based on multi-omics analysis. J China Pharmaceutical Univ. (2025) 56:634–44. 10.11665/j.issn.1000-5048.2025030301

[116] Park W Lim W Kim M Jang H Park SJ Song G. Female reproductive disease, endometriosis: from inflammation to infertility. Mol Cells. (2025) 48:100164. 10.1016/j.mocell.2024.100164 39617101 PMC11760828

[117] Crestani B Uccella S Pavone M Barra F Baggio S Ceccaroni M. Gut microbiota alterations and reproductive tract dysbiosis in endometriosis: a systematic review. Medicina (B Aires). (2026) 62:351. 10.3390/medicina62020351 PMC12942269 41752750

[118] Pérez-Prieto I Vargas E Salas-Espejo E Lüll K Canha-Gouveia A Pérez LA. Gut microbiome in endometriosis: a cohort study on 1000 individuals. BMC Med. (2024) 22:294. 10.1186/s12916-024-03503-y 39020289 PMC11256574

[119] Nannini G Cei F Amedei A. Unraveling the contribution of estrobolome alterations to endometriosis pathogenesis. Curr Issues Mol Biol. (2025) 47:502. 10.3390/cimb47070502 40728971 PMC12293932

[120] Yuanyue L Dimei O Ling L Dongyan R Xiaomei W. Association between endometriosis and gut microbiota: systematic review and meta-analysis. Front Microbiol. (2025) 16:1552134. 10.3389/fmicb.2025.1552134 40400684 PMC12092452

[121] Kobayashi H. Similarities in pathogenetic mechanisms underlying the bidirectional relationship between endometriosis and pelvic inflammatory disease. Diagnostics. (2023) 13:868. 10.3390/diagnostics13050868 36900012 PMC10000848

[122] Qiao L Zhao D Wu J. Evaluation of microecological therapy in endometriosis through modulation of the gut microbiota. Eur J Med Res. (2025) 30:1084. 10.1186/s40001-025-03334-4 41204376 PMC12595740

[123] Rostami S Alyasin A Saedi M Nekoonam S Khodarahmian M Moeini A. Astaxanthin ameliorates inflammation, oxidative stress, and reproductive outcomes in endometriosis patients undergoing assisted reproduction: a randomized, triple-blind placebo-controlled clinical trial. Front Endocrinol. (2023) 14:1144323. 10.3389/fendo.2023.1144323 PMC10067663 37020589

[124] Varney JE So D Gibson PR Rhys-Jones D Lee YSJ Fisher J. Clinical trial: effect of a 28-day low FODMAP diet on gastrointestinal symptoms associated with endometriosis (EndoFOD)—a randomised, controlled crossover feeding study. Aliment Pharmacol Ther. (2025) 61:1889–903. 10.1111/apt.70161 40319391 PMC12107219

[125] Kralj S Zeman K Mikuš M Karačić A Pršo A-ML Ćorić M. The role of probiotics in the treatment of endometriosis (ProMetrioS): a randomized double-blinded placebo-controlled cross-over trial. Trials. (2026) 27:242. 10.1186/s13063-025-09405-5 41715206 PMC13032410

[126] Radboud University Medical Center. Pain in Endometriosis and the Relation to Lifestyle: Effectiveness of a Dietary Intervention and Cognitive Behavioral Therapy in Endometriosis-Associated Pain. ClinicalTrials.gov Identifier: NCT06332560. Available online at: https://clinicaltrials.gov/study/NCT06332560 (Accessed June 25, 2026).

[127] Alghetaa H Mohammed A Singh NP Bloomquist RF Chatzistamou I Nagarkatti M. Estrobolome dysregulation is associated with altered immunometabolism in a mouse model of endometriosis. Front Endocrinol. (2023) 14:1261781. 10.3389/fendo.2023.1261781 PMC10748389 38144564

[128] Escorcia Mora P Valbuena D Diez-Juan A. The role of the gut Microbiota in female reproductive and gynecological health: insights into endometrial signaling pathways. Life. (2025) 15:762. 10.3390/life15050762 40430189 PMC12113314

[129] Liu X Wang Y Wen X Hao C Ma J Yan L. Platelet rich plasma alleviates endometritis induced by lipopolysaccharide in mice via inhibiting TLR4/NF-κB signaling pathway. Am J Reprod Immunol. (2024) 91:e13833. 10.1111/aji.13833 38467595

[130] Zhou F Zhao F Huang Q Lin X Zhang S Dai Y. NLRP3 Activated macrophages promote endometrial stromal cells migration in endometriosis. J Reprod Immunol. (2022) 152:103649. 10.1016/j.jri.2022.103649 35714422

[131] Wu Q-R Yang H Zhang H-D Cai Y-J Zheng Y-X Fang H. IP3R2-mediated Ca2+ release promotes LPS-induced cardiomyocyte pyroptosis via the activation of NLRP3/Caspase-1/GSDMD pathway. Cell Death Discov. (2024) 10:91. 10.1038/s41420-024-01840-8 38378646 PMC10879485

[132] Zhang M Shi Z Peng X Cai D Peng R Lin Y. NLRP3 inflammasome-mediated pyroptosis induce Notch signal activation in endometriosis angiogenesis. Mol Cell Endocrinol. (2023) 574:111952. 10.1016/j.mce.2023.111952 37268099

[133] Wu T Guo Y. ATP/P2x4 regulates inflammation and oxidative stress in endometriosis through NLRP3 inflammasome–dependent mechanisms. American J Rep Immunol. (2025) 94:e70132. 10.1111/aji.70132 40815040

[134] Ma S Zheng L Zhuang X Wang M Zou Y. Pathogenic mechanisms and therapeutic potential of the microbiome in premature ovarian insufficiency. Front Immunol. (2025) 16:1734367. 10.3389/fimmu.2025.1734367 41476963 PMC12748176

[135] Huang X Xu R Yang Q Jiang X Lin J Zhao H. The depletion of gut microbiome impairs the beneficial effect of Gui-Shen-Wan in restoring mice ovarian function and associated protein expression of ovarian tissues. Front Cell Infect Microbiol. (2024) 14:1505958. 10.3389/fcimb.2024.1505958 39664494 PMC11632464

[136] Cao J Ma W Chang X Pu D Tan R Hu L. Probiotics may improve vaginal microbiota, metabolic disorders and ovarian function-related markers by modulating gut microbiota in POI mice. BMC Microbiol. (2025) 25:375. 10.1186/s12866-025-04097-y 40597619 PMC12218955

[137] Munyoki SK Goff JP Reshke A Wilderoter E Mafarachisi N Kolobaric A. The microbiota extends the reproductive lifespan of mice by safeguarding the ovarian reserve. Cell Host Microbe. (2025) 33:1731–1747.e8. 10.1016/j.chom.2025.09.006 41005310 PMC13374525

[138] Liu S Wang T Liu Y Li Y Ma J Hu Q. Human placental mesenchymal stem cells ameliorates premature ovarian insufficiency via modulating gut microbiota and suppressing the inflammation in rats. PLoS One. (2025) 20:e0313763. 10.1371/journal.pone.0313763 40043087 PMC11882084

[139] Sreerangaraja Urs DB Wu W-H Komrskova K Postlerova P Lin Y-F Tzeng C-R. Mitochondrial function in modulating human Granulosa cell steroidogenesis and female fertility. Int J Mol Sci. (2020) 21:3592. 10.3390/ijms21103592 32438750 PMC7279321

[140] Cai Y Yang H Xu H Li S Zhao B Wang Z. β-nicotinamide mononucleotide reduces oxidative stress and improves steroidogenesis in granulosa cells associated with sheep prolificacy via activating AMPK pathway. Antioxidants. (2024) 14:34. 10.3390/antiox14010034 39857368 PMC11762531

[141] Yu Y Shan Y Lu J Xian Y Tang Z Guo X. Ferroptosis and sterol biosynthesis dysregulation in granulosa cells of patients with diminished ovarian reserve. Antioxidants. (2025) 14:749. 10.3390/antiox14060749 40563381 PMC12189052

[142] Liu L Li X Chen Y Li YZ Liu Z Duan Y. Interleukin-22 promotes proliferation and reverses LPS-induced apoptosis and steroidogenesis attenuation in human ovarian granulosa cells: implications for polycystic ovary syndrome pathogenesis. J Matern Fetal Neonatal Med. (2023) 36:2253347. 10.1080/14767058.2023.2253347 37661176

[143] Ju W Zhao Y Yu Y Zhao S Xiang S Lian F. Mechanisms of mitochondrial dysfunction in ovarian aging and potential interventions. Front Endocrinol. (2024) 15:1361289. 10.3389/fendo.2024.1361289 PMC11061492 38694941

[144] Zhao H Wang Y Han H Jiang Y Ji X Zhang Y. The role of mitophagy in female reproductive system diseases: from molecular mechanisms to therapeutic strategies. Front Endocrinol. (2025) 16:1645711. 10.3389/fendo.2025.1645711 PMC12668967 41341128

[145] Pan P Cao S Gao H Qu X Ma Y Yang J. Immp2l gene knockout induces granulosa cell senescence by activation of cGAS-STING pathway via TFAM-mediated mtDNA leakage. Int J Biol Macromol. (2025) 307:142368. 10.1016/j.ijbiomac.2025.142368 40120895

[146] Su D Ma R Su H Tan C Zhu Y Liu Y. Hydroxychloroquine alleviates cyclophosphamide-induced premature ovarian failure by attenuating granulosa cell senescence and modulating the mtDNA-cGAS pathway. npj Aging. (2026) 12:63. 10.1038/s41514-026-00359-9 41839900 PMC13139422

[147] D’Amico F Perrone AM Rampelli S Coluccelli S Barone M Ravegnini G. Gut microbiota dynamics during chemotherapy in epithelial ovarian cancer patients are related to therapeutic outcome. Cancers. (2021) 13:3999. 10.3390/cancers13163999 34439153 PMC8393652

[148] Zhao T Wang X Fu L Yang K. Fusobacterium nucleatum: a new player in regulation of cancer development and therapeutic response. Cancer Drug Resist. (2022) 5:436–50. 10.20517/cdr.2021.144 35800370 PMC9255244

[149] Chen W Chen X Fang Y Sun Y Lin Y. Research progress of probiotics intervention on reconstruction of intestinal flora and improvement of quality of life in patients after endometrial cancer surgery. Front Cell Infect Microbiol. (2025) 15:1670836. 10.3389/fcimb.2025.1670836 41050754 PMC12491236

[150] Lin A Huang L Jiang A Zhu L Mou W Li Y. Microbiota boost immunotherapy? A meta-analysis dives into fecal microbiota transplantation and immune checkpoint inhibitors. BMC Med. (2025) 23:341. 10.1186/s12916-025-04183-y 40484955 PMC12147380

[151] Chambers L. A randomized, double-blind, placebo-controlled study to investigate efficacy of a probiotic intervention on the gut and vaginal microbiome of ovarian cancer patients undergoing treatment with platinum chemotherapy. ClinicalTrials.gov Identifier: NCT07144826. Available online at: https://clinicaltrials.gov/study/NCT07144826 (Accessed June 25, 2026). 10.1016/j.ygyno.2026.06.016 42364424

[152] Chalif J Morton M Haight P Mehra Y O’Malley D Spakowicz D. Assessment of probiotic and prebiotic use in gynecologic cancer patients: a systematic review. Am J Obstet Gynecol. (2026) 234:893–918. 10.1016/j.ajog.2025.09.042 41072704

[153] Hu X Li B Li Y Liang Y Huang T. Communication between gut microbiota-derived metabolites and the tumor microenvironment. Front Immunol. (2025) 16:1649438. 10.3389/fimmu.2025.1649438 41169397 PMC12568434

[154] Liu M Fu X Yi Q Xu E Dong L. Impaired mitochondrial oxidative phosphorylation induces CD8+ T cell exhaustion. Biochem Biophys Res Commun. (2024) 734:150738. 10.1016/j.bbrc.2024.150738 39342799

[155] Kumar S Mittal S Gupta P Singh M Chaluvally-Raghavan P Pradeep S. Metabolic reprogramming in tumor-associated macrophages in the ovarian tumor microenvironment. Cancers. (2022) 14:5224. 10.3390/cancers14215224 36358644 PMC9656653

[156] Tábara L-C Segawa M Prudent J. Molecular mechanisms of mitochondrial dynamics. Nat Rev Mol Cell Biol. (2025) 26:123–46. 10.1038/s41580-024-00785-1 39420231

[157] Larnder AH Manges AR Murphy RA. The estrobolome: estrogen-metabolizing pathways of the gut microbiome and their relation to breast cancer. Int J Cancer. (2025) 157:599–613. 10.1002/ijc.35427 40177842 PMC12178105

[158] Raudenska M Balvan J Zgarbova E Kalfert D Plzak J Masarik M. Shaping death: how the microbiome regulates tumour cell demise and therapy response. Cancer Metastasis Rev. (2026) 45:9. 10.1007/s10555-026-10318-1 41714812 PMC12920367

[159] Alshehri B. Cytochrome c and cancer cell metabolism: a new perspective. Saudi Pharm J. (2024) 32:102194. 10.1016/j.jsps.2024.102194 39564377 PMC11570848

[160] Xie X-Q Yang Y Wang Q Liu H-F Fang X-Y Li C-L. Targeting ATAD3A-PINK1-mitophag*y* axis overcomes chemoimmunotherapy resistance by redirecting PD-L1 to mitochondria. Cell Res. (2023) 33:215–28. 10.1038/s41422-022-00766-z 36627348 PMC9977947

[161] Xia L Yan X Zhang H. Mitochondrial DNA-activated cGAS-STING pathway in cancer: mechanisms and therapeutic implications. Biochim Biophys Acta Rev Cancer. (2025) 1880:189249. 10.1016/j.bbcan.2024.189249 39701325

[162] Aloraini GS. Mitochondrial DNA release and cGAS-STING activation: emerging insights into anti-tumor immunity. Pathol Res Pract. (2025) 273:156158. 10.1016/j.prp.2025.156158 40774059

[163] Xu H Liu X Sun W Dong X Liu X Xie Y. Multi-omics elucidation of lactiplantibacillus plantarum NKK20 in preventing PCOS via the gut-ovar*y* axis: SCFAs-mediated microbiota-metabolite-immune crosstalk. Front Nutr. (2026) 12:1709581. 10.3389/fnut.2025.1709581 41624200 PMC12853653

[164] Dong X Xie F Li P. Modulation of gut microbiota and short-chain fatty acids by probiotics attenuates inflammation in endometriosis. Front Microbiol. (2026) 16:1713258. 10.3389/fmicb.2025.1713258 41704847 PMC12908314

[165] Geng L Yang X Sun J Ran X Zhou D Ye M. Gut microbiota modulation by inulin improves metabolism and ovarian function in polycystic ovary syndrome. Adv Sci. (2025) 12:2412558. 10.1002/advs.202412558 PMC12120758 40192074

[166] Cavaliere G Catapano A Trinchese G Cimmino F Penna E Pizzella A. Butyrate improves neuroinflammation and mitochondrial impairment in cerebral cortex and synaptic fraction in an animal model of diet-induced obesity. Antioxidants. (2022) 12:4. 10.3390/antiox12010004 36670866 PMC9854835

[167] Martinelli S Nannini G Cianchi F Staderini F Coratti F Amedei A. Microbiota transplant and gynecological disorders: the bridge between present and future treatments. Microorganisms. (2023) 11:2407. 10.3390/microorganisms11102407 37894065 PMC10609601

[168] Leong KSW Jayasinghe TN Wilson BC Derraik JGB Albert BB Chiavaroli V. Effects of fecal microbiome transfer in adolescents with obesity: the gut bugs randomized controlled trial. JAMA Netw Open. (2020) 3:e2030415. 10.1001/jamanetworkopen.2020.30415 33346848 PMC7753902

[169] Zhao M Chen D Hu X Xie C Xu L Zhou F. Gut-ovar*y* axis in polycystic ovary syndrome: mechanistic insights and gut microbiota-targeted therapeutic strategies. Front Endocrinol. (2025) 16:1684492. 10.3389/fendo.2025.1684492 PMC12626779 41268164

[170] Yadegar A Bar-Yoseph H Monaghan TM Pakpour S Severino A Kuijper EJ. Fecal microbiota transplantation: current challenges and future landscapes. Clin Microbiol Rev. (2024) 37:e00060-22. 10.1128/cmr.00060-22 38717124 PMC11325845

[171] Salahi E Amidi F Zahiri Z Aghahosseini M Mashayekhi F Abkenari S. The effect of mitochondria-targeted antioxidant MitoQ10 on redox signaling pathway components in PCOS mouse model. Arch Gynecol Obstet. (2021) 305:1–10. 10.1007/s00404-021-06230-4 34633506

[172] Castelôa M Moreira-Pinto B Benfeito S Borges F Fonseca BM Rebelo I. *In Vitro* effects of mitochondria-targeted antioxidants in a small-cell carcinoma of the ovary of hypercalcemic type and in type 1 and type 2 endometrial cancer. Biomedicines. (2022) 10:800. 10.3390/biomedicines10040800 35453550 PMC9030827

[173] Vinten KT Trętowicz MM Coskun E van Weeghel M Cantó C Zapata-Pérez R. NAD+ precursor supplementation in human ageing: clinical evidence and challenges. Nat Metab. (2025) 7:1974–90. 10.1038/s42255-025-01387-7 41083806

[174] Fan Y Hu Q Wu X Lang P Wei Y Yi R. Reverse effects of nicotinamide mononucleotide supplementation on declining quality of oocytes with polycystic ovary syndrome. FASEB J. (2025) 39:e70846. 10.1096/fj.202500921R 40678956 PMC12272325

[175] Zhang C Song Y Chen L Chen P Yuan M Meng Y. Urolithin A attenuates hyperuricemic nephropathy in fructose-fed mice by impairing STING-NLRP3 axis-mediated inflammatory response via restoration of parkin-dependent mitophagy. Front Pharmacol. (2022) 13:907209. 10.3389/fphar.2022.907209 35784701 PMC9240289

[176] Mishra SR Mishra P Senapati PK Mahapatra KK Bhutia SK. Intricate role of DRP1 and associated mitochondrial fission signaling in carcinogenesis and cancer progression. Biochim Biophys Acta Rev Cancer. (2025) 1880:189453. 10.1016/j.bbcan.2025.189453 40967345

[177] Chen L Zhang L Hua H Liu L Mao Y Wang R. Interactions between toll-like receptors signaling pathway and gut microbiota in host homeostasis. Immun Inflamm Dis. (2024) 12:e1356. 10.1002/iid3.1356 39073297 PMC11284964

[178] McWilliams TG Muqit MM. PINK1 and Parkin: emerging themes in mitochondrial homeostasis. Curr Opin Cell Biol. (2017) 45:83–91. 10.1016/j.ceb.2017.03.013 28437683

[179] Puri G Naura AS. Implication of mitochondrial ROS-NLRP3 inflammasome axis during two-hit mediated acute lung injury in mice. Free Radical Res. (2022) 56:1–16. 10.1080/10715762.2021.2023740 35129032

[180] Macleod M Pradana F Wadley AJ Barlow J. Comprehensive real-time metabolic profiling of peripheral blood mononuclear cells reveals important methodological considerations for immunometabolism research. Front Immunol. (2025) 16:1676550. 10.3389/fimmu.2025.1676550 41235236 PMC12605198

[181] Su B Cao Y Ma L Huang J. BMI-stratified phenotypes of polycystic ovary syndrome: advances in gut microbiota research and personalized management strategies. Front Endocrinol. (2026) 17:1734041. 10.3389/fendo.2026.1734041 PMC12920190 41727680

[182] Zmora N Suez J Elinav E. You are what you eat: diet, health and the gut microbiota. Nat Rev Gastroenterol Hepatol. (2019) 16:35–56. 10.1038/s41575-018-0061-2 30262901

[183] Shahid U. Microbiome-guided precision medicine: mechanistic insights, multi-omics integration, and translational horizons. J Precision Med Health Dis. (2025) 3:100018. 10.1016/j.premed.2025.100018

[184] Zhang L Zhao J Cheng G Wu J Liu Y Lei G. Inflammasomes meet organoids and artificial intelligence: unraveling the complexity of gynecological inflammation. Front Immunol. (2026) 17:1753651. 10.3389/fimmu.2026.1753651 42079573 PMC13132712

[185] See J-E Barlow S Arjumand W DuBose H Segato Dezem F Plummer J. Spatial omics: applications and utility in profiling the tumor microenvironment. Cancer Metastasis Rev. (2025) 44:87. 10.1007/s10555-025-10304-z 41331191 PMC12672822

[186] Williams EC Franzén L Olsson Lindvall M Hamm G Oag S Majumder MM. Spatially resolved integrative analysis of transcriptomic and metabolomic changes in tissue injury studies. Nat Commun. (2026) 17:205. 10.1038/s41467-025-68003-w 41501078 PMC12780049

[187] Qi Y Chen X Zheng S Wu T Li Z Cheng J. Single-cell and spatially resolved omics reveal transcriptional and metabolic signatures of ovarian endometriomas. Nat Commun. (2025) 16:11539. 10.1038/s41467-025-66706-8 41274870 PMC12749035

[188] Xu Z Yeoh YK Tun HM Fei N Zhang J Morrison M. Variation in the metagenomic analysis of fecal microbiome composition calls for a standardized operating approach. Microbiol Spectr. (2024) 12:e01516-24. 10.1128/spectrum.01516-24 39475247 PMC11619352

[189] Knight R Vrbanac A Taylor BC Aksenov A Callewaert C Debelius J. Best practices for analysing microbiomes. Nat Rev Microbiol. (2018) 16:410–22. 10.1038/s41579-018-0029-9 29795328

[190] Yin X-F Ye T Chen H-L Liu J Mu X-F Li H. The microbiome compositional and functional differences between rectal mucosa and feces. Microbiol Spectr. (2024) 12:e03549-23. 10.1128/spectrum.03549-23 38916335 PMC11302734

[191] Hatcher C Richenberg G Waterson S Nguyen LH Joshi AD Carreras-Torres R. Application of Mendelian randomization to explore the causal role of the human gut microbiome in colorectal cancer. Sci Rep. (2023) 13:5968. 10.1038/s41598-023-31840-0 37045850 PMC10097673

